# Methylglyoxal detoxifying gene families in tomato: Genome-wide identification, evolution, functional prediction, and transcript profiling

**DOI:** 10.1371/journal.pone.0304039

**Published:** 2024-06-12

**Authors:** Abdullah Al Masum, Md Sakil Arman, Ajit Ghosh

**Affiliations:** Department of Biochemistry and Molecular Biology, Shahjalal University of Science and Technology, Sylhet, Bangladesh; South Asian University, INDIA

## Abstract

Methylglyoxal (MG) is a highly cytotoxic molecule produced in all biological systems, which could be converted into non-toxic D-lactate by an evolutionarily conserved glyoxalase pathway. Glutathione-dependent glyoxalase I (GLYI) and glyoxalase II (GLYII) are responsible for the detoxification of MG into D-lactate in sequential reactions, while DJ-1 domain containing glyoxalase III (GLYIII) catalyzes the same reaction in a single step without glutathione dependency. Afterwards, D-lactate dehydrogenase (D-LDH) converts D-lactate into pyruvate, a metabolically usable intermediate. In the study, a comprehensive genome-wide investigation has been performed in one of the important vegetable plants, tomato to identify 13 putative GLYI, 4 GLYII, 3 GLYIII (DJ-1), and 4 D-LDH genes. Expression pattern analysis using microarray data confirmed their ubiquitous presence in different tissues and developmental stages. Moreover, stress treatment of tomato seedlings and subsequent qRT-PCR demonstrated upregulation of *SlGLYI-2*, *SlGLYI-3*, *SlGLYI-6A*, *SlGLYII-1A*, *SlGLYII-3B*, *SlDJ-1A*, *SlDLDH-1* and *SlDLDH-4* in response to different abiotic stresses, whereas *SlGLYI-6B*, *SlGLYII-1B*, *SlGLYII-3A*, *SlDJ-1D* and *SlDLDH-2* were downregulated. Expression data also revealed *SlGLYII-1B*, *SlGLYI-1A*, *SlGLYI-2*, *SlDJ-1D*, and *SlDLDH-4* were upregulated in response to various pathogenic infections, indicating the role of MG detoxifying enzymes in both plant defence and stress modulation. The functional characterization of each of these members could lay the foundation for the development of stress and disease-resistant plants promoting sustainable agriculture and production.

## Introduction

The effects of climate change due to global warming result in various forms of environmental stress, dramatically decreasing crop yield and declining economic development. During adverse conditions, cytotoxic methylglyoxal (MG) is produced in biological systems through several non-enzymatic reactions as a byproduct of carbohydrate, protein and fatty acid metabolism [1]. MG introduces toxicity by generating free radicals and advanced glycation end-products (AGEs), thus reducing growth as well as productivity [2]. Dicarbonyl methylglyoxal is a renowned mutagen and genotoxic reactive, capable of altering protein and nucleic acid. Any modification of arginine’s guanidine groups in protein causes the production of AGEs, which cause MG-induced damage in biological systems [2]. Imidazopurinone derivatives, the most common AGEs generated from MG, have been linked to genomic integrity loss and genotoxicity [3]. In response to the herbicide imazethapyr, crops such as lentils accumulate methylglyoxal, detoxified by glyoxalase pathway enzymes [4].

**Fig 1:**
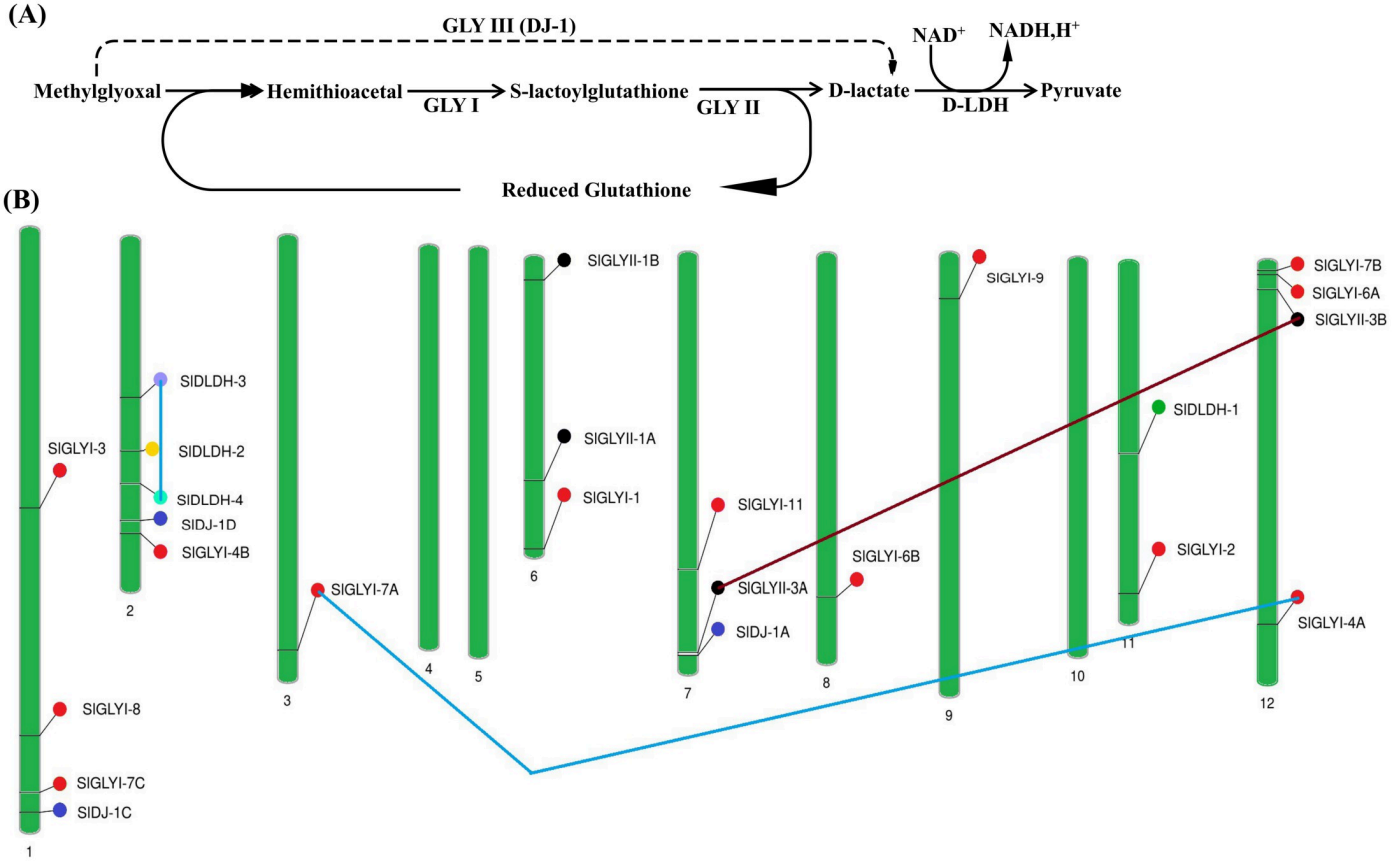
Role of methylglyoxal detoxifying members in plants and their chromosomal location in tomato. (A) Schematic diagram of the glyoxalase route for methylglyoxal (MG) detoxification in plants. In the glutathione-dependent pathway, MG is converted to S-D-lactotyl glutathione and D-lactate sequentially by GLYI and GLYII. GLYIII catalyzes the reaction in a single step without the aid of reduced glutathione. D-lactate is then converted to pyruvate by CYTc-dependent D lactate dehydrogenase (DLDH). (B) Chromosomal distribution of glyoxalases and DLDH genes in *Solanum lycopersicum*. The presence and position of all identified genes were shown in nine different chromosomes with three segmental duplication events added by a straight line.

The glyoxalase pathway, responsible for detoxifying MG, is highly conserved and ubiquitous among lower prokaryotes as well as higher eukaryotes such as humans and plants [5]. Two conventional glyoxalase enzymes, glyoxalase I (GLYI) and glyoxalase II (GLYII), convert MG derivative hemithioacetal (HTA) into D-lactate through two sequential reactions in a glutathione-dependent pathway [6]. By contrast, a unique glyoxalase enzyme, a homolog to DJ-1 proteins in *Arabidopsis*, glyoxalase III (GLYIII) catalyzes the whole process in a single step in a glutathione-independent manner [7–9] (Fig 1A). In the final step of MG detoxification, TCA cycle metabolite pyruvate is formed from D-lactate by the action of D-lactate dehydrogenase (D-LDH) [10].

Glyoxalases had been studied previously in several monocotyledons and dicotyledons, including *Zea mays*, *Corchorus capsularis*, *Triticum*, *Aloe vera*, *Glycine max*, *Spinacia oleracea*, *Cicer*, *Nicotiana*, *Brassica*, *Pisum sativum*, *Amaranthus* and *Sorghum bicolor* [5, 11–14]. However, in comparison, microbial systems and humans have undergone more comprehensive studies and characterization of GLYI and GLYII enzymes for their co-factor dependencies. Plants GLYI require either Zn^2+^ or Ni^2+^ as co-factors, while GLYIII was found to have no metal ion dependencies [15, 16]. D-LDH, on the other hand, showed dependency on cytochrome c or NAD and falls into the category of either D-lactate ferricytochrome c oxidoreductase or D-lactate NAD oxidoreductase [10, 17].

The glyoxalase system is significant in maintaining the normal homeostasis of plants by preventing any alteration of molecular and morphological characteristics upon exposure to stress [6]. It has been thoroughly investigated because of its capacity to protect against harmful environmental conditions by detoxifying MG, which would otherwise cause significant hazards under diverse abiotic and biotic stresses [18]. The role of glyoxalase in transgenic plants confirmed its function in abiotic stress modulation. Overexpression of the *GLYI* or *GLYII* gene showed MG and salt tolerance in the transgenic tobacco [19, 20]. Transgenic tobacco lines overexpressing both *GLYI* and *GLYII*, on the other hand, showed greater tolerance to salt stress [19]. *Brassica juncea* exhibited a considerable increase in *GLYI* expression against salt, mannitol, and heavy metal stress [19]. Similarly, the GLYI members were substantially expressed in *Manihot esculenta* (cassava) in response to iron-induced stress, whereas the overexpression of *MeGLYI-13* confers iron toxicity tolerance in engineered *Arabidopsis* [21]. When exposed to several abiotic stressors, the rice *GLYII* gene showed early activation, including NaCl, evaporation, heat, and abscisic or salicylic acid treatments [22]. Biotic stress related to *Agrobacterium tumefaciens* infectious altered glyoxalase expression patterns [23]. *GLYI* genes had lower expression in rice after being infected with *Xanthomonas oryzae pv*. *Oryzae* or *Pyricularia grisea* [24].

Substantial research has been conducted on the glyoxalase pathway in various species of microbes. However, only a few plant species had been analyzed genome-wide to identify MG detoxifying gene family members. Tomato is an economically important crop cultivated worldwide. According to FAOSTAT of the United Nations, about 186,821,216 metric tonnes of tomatoes were produced worldwide in 2020 [25]. However, the production suffered from exposure to adverse weather and pathogenic infection. Therefore, a genome-wide investigation has been conducted to evaluate the significance of glyoxalase and D-LDH members in the stress physiology of tomato, which had identified a total of 13 GLYI, 4 GLYII, 3 GLYIII, and 4 D-LDH genes. Expression profiling and enzyme activity of these families reveal their developmental and environmental influences. This study will pave the foundation to explore further the physiological roles of these members in MG detoxification as well as stress modulation of plants.

## Materials and methods

### Identification and nomenclature of putative glyoxalase and D-lactate dehydrogenase members in tomato

To identify MG detoxifying members in tomato, previously characterized *Arabidopsis* glyoxalases (e.g. AT1G07645, AT1G06130, AT3G02720 etc.) and D-lactate dehydrogenase (e.g. AT4G36400, AT4G36400 etc.) proteins [9, 17, 26] were used as queries for Blastp search (e value: -1) against tomato genome ‘*Solanum lycopersicum ITAG3*.*2*’ in Phytozome v13 database [27]. The resulting protein sequences were then analyzed using Pfam (e value: 1) [28] to confirm the presence of HMM profile PF00903, PF00753, PF01965.19 and PF01565 for GLYI, GLYII, GLYIII, and D-LDH, respectively. InterProScan [29, 30] and SMART [31, 32] were used to recheck the existence of the essential domains. The identified members were designated based on the sequence similarity with *Arabidopsis* orthologues by following the Arabic number system in alphabetic order and denoted by the prefix “Sl” for *Solanum lycopersicum*. Protein features of the identified members were predicted using the expassy protparam tools [33]. The Plant-PLoc server [34] was utilized to determine the subcellular localization of the proteins.

### Prediction of chromosomal localization and gene duplication events

Using information from the Phytozome Database, the locations of all newly identified glyoxalases and D-lactate dehydrogenases were mapped onto the twelve chromosomes of the tomato. Duplication occurrences were predicted using Blastp search (e-value 10^−10^) in phytozome with 80% sequence identity. Tandemly duplicated (TD) genes were classified as those that included more than one homologous gene inside a 100 kilobase pair region on the same chromosome, whereas segmental duplicated (SD) genes were characterized as those that were not contained within the 100-kb region. The rate of synonymous substitution (dS) and non-synonymous substitution (dN), as well as evolutionary pressure (dN/dS) between the duplicated glyoxalase and D lactate dehydrogenase gene pairs, were determined by the PAL2NAL tool [35]. Each duplicated gene pair’s divergence period (T) was measured by T = dS/2λ, with a constant rate of 1.5108 substitutions per site per year for dicotyledonous plants.

### Multiple sequence alignment and phylogenetic analysis of MG detoxifying members

Complete amino acid sequences of the putative glyoxalases and D-lactate dehydrogenases were aligned with their known orthologues from diverse plant species using ClustalW to identify the evolutionary relationship. Jalview 2 [36] was used to visualize the alignment. The evolutionary relationship and divergence of proteins were observed through the phylogenetic tree, built by MEGA-X [37], following the Neighbourhood-joining method with 1000 bootstraps. iTOL [38] was used for the modification and visualization of the tree.

### Genomic structure analysis and protein architecture of the identified members

The exon-intron structures of glyoxalase and D-LDH genes were analyzed by comparing genomic and coding DNA sequences (CDS) through the Gene Structure Display Server (GSDS 2.0) [39]. Gene features such as UTR, exon-intron, and intron phase locations were extracted from the GSDS server. Pfam and SMART were utilized to identify the domain positions and extract the amino acid sequences for the respective domains. Schematic representations of domain organization were manually created, and tables summarizing the presence of conserved residues within these domains were compiled.

### Homology modeling of representative glyoxalase and D-LDH proteins in tomato

The three-dimensional structures of putative active SlGLYI-2, SlGLYI-3, SlGLYII-1A, SlDJ-1A, and SlDLDH-1 were predicted using SWISS-MODEL [40] by utilizing experimentally resolved crystal structure from Protein Data Bank (PDB) as templates, selected by the highest sequence similarity. The homology model for SlGLYI-2 was constructed using the *Gossypium hirsutum* glyoxalase (PDB ID: 7VQ6) with 55% sequence similarity. Likewise, models for SlGLYI-3 and SlGLYII-1A were based on *Zea mays* glyoxalase I (5D7Z, 54% similarity) and *Arabidopsis* AtGLYII-3 (2Q42, 56% similarity), respectively. SlDJ-1A was modelled after the AtDJ-1D structure (4OFW, 31% similarity), and SlDLDH-1 was based on the crystal structure of mouse mitochondrial mDLDH (8JDE, 42% similarity). The active site residues in the predicted structures were determined by template analysis and mapped for functional insights. UCSF ChimeraX (version 1.7.1) was used to visualize and annotate the three-dimensional structures [41].

### Analysis of expression pattern of putative glyoxalases and D-LDH genes

Microarray data for tissue-specific expression of glyoxalases and D lactate dehydrogenase transcripts during various developmental stages of tomato were obtained through the Genevestigator Affymetrix tomato genome array database [42]. Normalized Expression profile was obtained for seventeen different tissue types, including underground, aerial and reproductive tissue, as well as six development stages of vegetative growth and maturation- “shoot growth, inflorescence, flowering, fruit formation, ripening and complete ripening”. Expression patterns in response to different biotic (pathogenic infection) and abiotic stressors (drought, salinity, heat) were retrieved from the same source. Genevestigator was used to calculate the log2 fold change ratio of each transcript under stress conditions as compared to the respective control. Heatmap was generated from the normalized expression data using the MeV software package [43] with the Manhattan distance correlation metrics [44].

### Analysis of cis-acting regulatory element in the promoter regions

The putative promoter sequence (1 kb upstream of the start codons) of tomato glyoxalase and D-lactate dehydrogenase genes was obtained from the Phytozome database and analyzed through PlantCARE web tools [45] to identify the presence and type of cis-acting regulatory elements. The results of the analysis were represented in a bar diagram. The function of cis-acting elements was determined through a literature review.

### Plant material and stress treatment

One of the Bangladeshi tomato varieties (BARI-4), seeds was collected from the Bangladesh Agricultural Research Institute (BARI), Bangladesh. Tomato seedlings were cultivated in a control condition with 14 hours of light and 8 hours of darkness at a temperature of 26±2°C. The 8-day-old seedlings were given a variety of treatments. 150 mM NaCl solution, 100 mM mannitol solution, and 5 mM H_2_O_2_ solution were used to mimic salinity, drought, and oxidative stress, respectively, while plants were kept in 4°C water or in an incubator at 40°C to impose the cold or heat stress, respectively. Plants were kept under normal conditions for control. Shoot tissues were harvested at 0 h, 6 h, 12 h, and 24 h after the stress was applied.

### Extraction of RNA extraction, and quantitative real-time PCR analysis

Total RNA was extracted using the 6 h post-treatment harvested frozen shoot samples using the manufacturer’s instruction (SV Total RNA Isolation System, Promega Corporation, USA). The isolated RNA was quantified using a Thermo Scientific NanoDrop instrument and the first-strand cDNA was synthesized by using 10 μg of RNA according to the GoScript^™^ Reverse Transcriptase protocol (Promega Corporation, USA). Selected gene-specific primers were designed using the Primer-BLAST (https://ncbi.nlm.nih.gov/tools/primer-blast) and synthesized from Macrogen (https://dna.macrogen.com) along with *SlEF1α* as a reference gene [46] (S1 Table). Real-time PCR was performed in the QUANTSTUDIO^®^3 REAL-TIME PCR SYSTEM with SYBR Green qPCR kits, and the fold change in expression for each gene at each condition was calculated using the 2^-ΔΔCt^ method [47]. The experiments were carried out thrice for each condition and treatment.

### Enzyme activity of GLYI and GLYII in response to different abiotic stresses

The total plant protein was extracted using the extraction buffer containing 0.1 M sodium phosphate buffer, pH 7.0, 50% glycerol, 16 mM MgSO_4_, 0.2 mM PMSF and 0.2% polyvinylpolypyrrolidone [19]; and quantified using Bradford method [48]. The activity of GLYI was measured in 0.1 M sodium phosphate buffer (pH 7.5) with 3.5 mm MG and 1.7 mm reduced glutathione (GSH) as substrate by taking readings at 240 nm for the formation of S-Lactoylglutathione (SLG) over 5 min [19]. Similarly, GLYII enzyme activity was measured by monitoring the reduction of SLG in 10 mm MOPS and 300 mM SLG [19]. The activity of both enzymes was presented as μmol/min/mg of total protein. All the kinetic experiments were performed three times and the data were represented as the mean ± standard deviation (n = 3).

### Ethics approval

All experimental research on plants, including the collection of plant materials has been used by following the relevant institutional, national, and international guidelines and legislation.

## Results

### Identification of glyoxalases and D-lactate dehydrogenase members in tomato

Genome-wide analysis in tomato identified 13 putative GLYI, 4 GLYII, 3 unique GLYIII (DJ-1) and 4 D-LDH genes. The HMM Profile search using the Pfam database (E value 1.0) for the glyoxalase domain led to the pinpointing of 13 potential SlGLYI proteins with different transcript lengths (Table 1). There was no alternatively spliced transcript. They were designated as *SlGLYI-1* to *SlGLYI-11* based on their chromosomal position and similarities with *Arabidopsis* counterparts. Similarly, a total of 4 GLYII genes and 3 unique DJ-1 genes were identified by profile HMM search. GLYII genes were assigned as *SlGLYII-1* to *SlGLYII-3* based on similarity score and chromosomal position, while GLYIII (DJ-1) genes were labelled as SlDJ-1A to SlDJ-1D by following the previous report [9].

**Table 1:** List of identified glyoxalases and D-lactate dehydrogenase members in tomato along with their detailed information and localization.

	Sl no	Gene Name	Locus Name	Protein variants	CDS coordinate (5’–3’)	Strand	Gene Length (bp)	CDS length (bp)	Protein			
									Length (aa)	MW (kDa)	pI	Localization
GLY-I	1	SlGLYI-1	Solyc06g075990.3	Solyc06g075990.3.1	47321149–47322092	+	943	414	137	15.26	6.07	Chloroplast
	2	SlGLYI-2	Solyc11g069040.3	Solyc11g069040.3.1	53898717–53914681	-	15964	558	185	20.71	5.25	Chloroplast
	3	SlGLYI-3	Solyc02g080630.3	Solyc02g080630.3.1	45369390–45375171	-	5781	882	293	32.85	5.95	Chloroplast
	4	SlGLYI-4A	Solyc12g042840.2	Solyc12g042840.2.1	59202522–59204319	+	1797	522	173	19.57	5.40	Chloroplast
	5	SlGLYI-4B	Solyc02g084410.3	Solyc02g084410.3.1	48080132–48081779	+	1647	573	190	21.81	4.92	Plasma membrane
	6	SlGLYI-6A	Solyc12g007310.2	Solyc12g007310.2.1	1700809–1705531	-	4722	1191	396	44.28	7.55	Cytoplasm
	7	SlGLYI-6B	Solyc08g066850.3	Solyc08g066850.3.1	55795848–55807041	+	11193	1083	360	39.90	5.12	Chloroplast
	8	SlGLYI-7A	Solyc03g116530.3	Solyc03g116530.3.1	67397152–67398567	-	1415	537	178	19.88	6.42	Nucleus
	9	SlGLYI-7B	Solyc12g006640.2	Solyc12g006640.2.1	1107881–1112108	-	4227	498	165	19.05	5.28	Chloroplast
	10	SlGLYI-7C	Solyc01g103590.3	Solyc01g103590.3.1	92047495–92048349	+	854	564	187	20.95	5.27	Nucleus
	11	SlGLYI-8	Solyc01g088010.1	Solyc01g088010.1.1	82726843–82727623	+	780	708	235	27.44	5.83	Chloroplast
	12	SlGLYI-9	Solyc09g082120.3	Solyc09g082120.3.1	68378453–68380192	-	1739	477	158	17.70	6.51	Plasma membrane
	13	SlGLYI-11	Solyc07g040950.3	Solyc07g040950.3.1	51321652–51326445	-	4793	603	200	22.32	7.06	Chloroplast
GLY-II	14	SlGLYII-1A	Solyc06g053310.3	Solyc06g053310.3.1	36142344–36151320	+	8976	990	329	36.15	8.62	Chloroplast
	15	SlGLYII-1B	Solyc05g009140.3	Solyc05g009140.3.1	3254997–3264277	-	9280	1461	486	54.5	5.70	Chloroplast
	16	SlGLYII-3A	Solyc07g061960.3	Solyc07g061960.3.1	64955426–64960885	+	5459	855	284	31.41	8.23	Chloroplast
	17	SlGLYII-3B	Solyc12g011430.2	Solyc12g011430.2.1	4254485–4257606	+	3121	855	284	31.45	6.54	Cytoplasm
GLY-III	18	SlDJ-1A	Solyc07g062610.3	Solyc07g062610.3.1	65434982–65439873	+	4891	1452	483	51.74	6.24	Chloroplast
	19	SlDJ-1C	Solyc01g108070.3	Solyc01g108070.3.1	95329060–95333719	+	4659	1212	403	43.02	8.44	Chloroplast
	20	SlDJ-1D	Solyc02g081400.3	Solyc02g081400.3.1	45939942–45944446	-	4504	1167	388	42.02	5.48	Chloroplast
D-LDH	1	SlDLDH-1	Solyc11g045390.2	Solyc11g045390.2.1	30898939–30942082	-	43143	1716	571	62.77	6.45	Plasma membrane
	2	SlDLDH-2	Solyc02g062430.3	Solyc02g062430.3.1	34571089–34581960	+	10871	1770	589	65.47	6.81	Cytoplasm
	3	SlDLDH-3	Solyc02g030170.3	Solyc02g030170.3.1	25733240–25735670	-	2430	1878	626	65.87	8.01	Cytoplasm
	4	SlDLDH-4	Solyc02g069490.3	Solyc02g069490.3.1	39925286–39928562	-	3276	1701	566	72.6	6.51	Cytoplasm

Abbreviations: CDS = Coding DNA sequence, MW = Molecular weight, bp = base pair, aa = Amino Acid, KDa = Kilo Dalton, pI = isoelectric point

Initial screening for D-LDH found 24 genes having FAD_binding _4 domains. While these proteins had a common FAD-binding region, their catalytic activity might differ due to distinct secondary domains. Therefore, the domain organization of all identified FAD-binding superfamily members was analyzed to identify D-LDH, which specifically has D-lactate catalytic activity. In a previous study on sorghum, proteins containing only FAD_binding _4 domain as well as an additional FAD-oxidase_C were recognized as D-lactate catalyzing D-LDH, based on multiple sequence alignment and phylogenetic analysis [49]. Thus, four D-LDH genes were predicted in our investigation encoding functionally active D-LDH, with two of them containing additional FAD-oxidase_C domain. They were numbered as SlDLDH-1-SlDLDH-4 (Table 1) according to blast hits against *Arabidopsis thaliana* D-LDH genes.

### A comprehensive analysis of the identified SlGLYI and SlGLYII, SlDJ-1 and SlDLDH family members

The identified glyoxalases and D-LDH members were examined further to ascertain their physiochemical characteristics. The CDS length of SlGLYI members ranged from 414 bp (*SlGLYI-1*) to 1191 bp (*SlGLYI-6A*). Subsequently, SlGLYI-6A was the largest protein of the SlGLYI family with a length of 336 aa and size of 44.43 kDa, while SlGLYI -1 was the smallest one with 173 aa in length and 44.43 KDa in size (Table 1). The isoelectric points (pI) of the SlGLYI family ranged from 4.92 (SlGLYI-4B) to 7.55 (SlGLYI-7 and SlGLYI-6A), indicating that cationic and anionic SlGLYI proteins could coexist simultaneously under certain physiological conditions. The bulk of SlGLYI proteins were localized in the chloroplast, with a few members detected in the plasma membrane, nucleus, mitochondria, and cytosol (Table 1).

Similarly, the coding DNA sequence of SlGLYII ranged from 723 bp (*SlGLYII-1B*) to 1461 bp (*SlGLYII-3A*), with an average length of 1040 bp. SlGLYII-1B was the largest member in terms of polypeptide length (486 aa) and molecular weight (54.5 kDa), while SlGLYII-3A was the smallest. Both the SIGLYI and SIGLYII protein families had positively and negatively charged proteins. Most SlGLYII proteins, like SlGLYI members, showed chloroplast localization except for SlGLYII-3B, which was found in the cytoplasm.

The physicochemical properties of all the identified GLYIII-like proteins (DJ-1) were also investigated as with traditional glyoxalase enzymes. The CDS length of *SlDJ-1* ranged from 1167 (*SlDJ-1D*) to 1452 bp (*SlDJ-1A*). The smallest SlDJ-1 member, SlDJ-1D, had a length of 388 aa with a weight of 42.02 kDa, while the largest, SlDJ-1A, was comprised of 483 aa residues with a molecular weight of 51.74 kDa. The pI value ranges from 5.48 to 8.44. The Ploc server predicted that all the identified members were located in the chloroplast.

Similarly, D-LDH had CDS lengths ranging from 1701 bp (*SlDLDH-4*) to 1878 bp (*SlDLDH-3*). The identified genes have a mean molecular weight of 66 kDa. The pI of the majority of the identified members is acidic, except SlDLDH-3, with a basic pI. Likewise, most of the proteins were found to be localized in the cytoplasm, except SlDLDH-1 in the plasma membrane.

### Chromosomal localization and gene duplication analysis

Glyoxalases and D-LDH genes were randomly dispersed throughout nine chromosomes of the tomato, whereas no genes were detected in the other three chromosomes (Fig 1B). On chromosome 2, there were a maximum of five methylglyoxal detoxifying members, followed by four each in chromosomes 1 and 12; three genes each in chromosomes 6 and 7; two genes in chromosome 11, and one gene each in chromosome 3, 8 and 9. Three *SlDJ-1* genes were found to be distributed irregularly on chromosomes 1, 2, and 7. The D- lactate dehydrogenase genes were split across two chromosomes, with three genes on chromosome 2 and one on chromosome 11 (Fig 1B).

Three duplication events were observed among the identified genes. Between *SlDLDH-3* and *SlDLDH-4*, there is a single duplication event on chromosome 2. A duplication between *SlGLI-7A* and *SlGLYI-4A* linked chromosomes 3 and 12, while another duplication between *SlGLYII-3A* and *SlGLYII-3B* linked chromosomes 12 and 7 (Fig 1B). To study the selection pressure on the duplicated SlGLYs and SlDLDH gene pairs, the value of non-synonymous (dN) and synonymous (dS) substitutions were estimated to discover whether the ratio is greater, less or equal to 1, indicating a positive, purifying and neutral selection respectively [50–52]. All of the discovered duplicated gene pairs had a dN/dS ratio below 1, suggesting that purifying selection may play a role in their evolution (S2 Table). Additionally, the divergence period for the duplicated gene pairs is estimated to be between 23.6 and 37.9 million years ago.

### Gene and protein structure of conventional glyoxalases

The Gene Structure Display Server (GSDS 2.0) was used to depict the exon-intron structure of the genes to get an insight into the association between gene structure and activity. The exon-intron patterns of the SlGLYI genes showed a high degree of variation. *SlGLYI-3*, *SlGLYI-6B*, and *SlGLYI-6A* all contained eight exons and distinct introns, but other members have different types of intron-exon organization (S1A Fig). Genes encoding for GLYI enzymes fell into two groups based on sequence homology that exhibited distinct structural properties within each cluster (S1A Fig). By contrast, *SlGLYII* genes did not have such distinctive exon-intron combinations. *SlGLYII-3* and *SlGLYII-4* genes possessed nine exons and eight introns, whereas SlGLYII-3A contained nine exons and eight. *SlGLYII-2* had 12 exons, the most of any SlGLYII gene, in both spliced versions (S1C Fig).

Domain architecture of putative SlGLYI elucidates that only the glyoxalase domain was present in all identified SlGLYI proteins (S1B Fig). Three GLYI proteins, notably SlGLYI-3, SlGLYI-6A, and SlGLYI-6B, included two PF00903 domains, whereas the remaining proteins contained only one. The variation in the length of the glyoxalase domain has been linked with metal dependency [53, 54]. Thus, domain organization patterns may be used to predict the metal ion dependencies of SlGLYI proteins (S1B Fig). Moreover, while all SlGLYII proteins had metallo-lactamase domains (S1D Fig), only SlGLYII-1A possessed an additional HAGH_C (PF01623) domain, which was found responsible for the hydrolysis D-lactoyl-glutathione to form glutathione and D-lactate in human [55]. Also, crystal structure analysis revealed that substrate binding might occur at the interface between HAGH_C and the catalytic β-lactamase region [56]. Further, all identified SlGLYII contained the active site motif (C/GHT) in addition to metallo-lactamase and thus was functionally active (S2B Fig).

### Phylogenetic relationship, multiple sequence alignment and functional prediction of conventional glyoxalases

**Fig 2:**
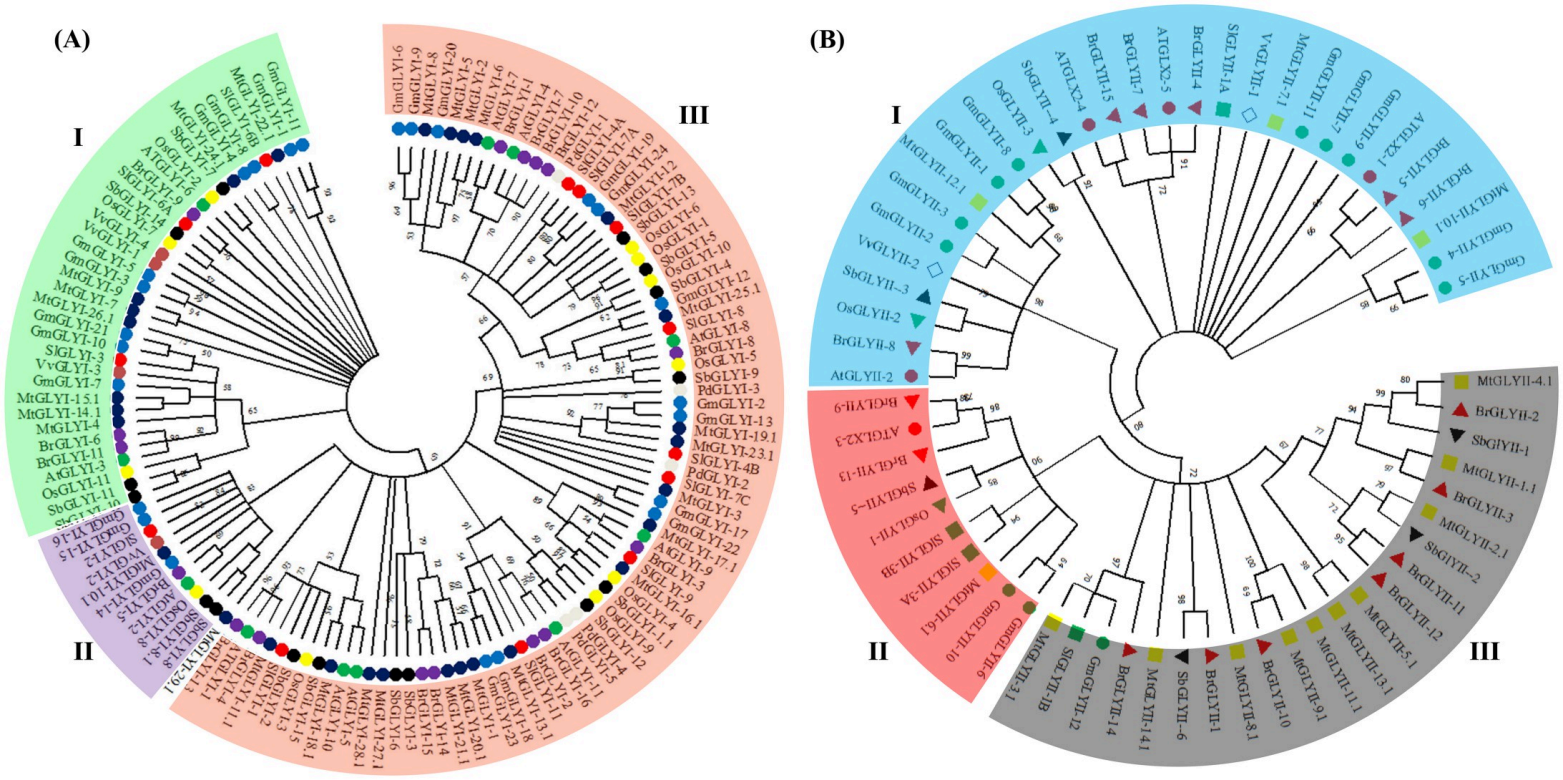
Evolutionary relationship of conventional glyoxalase proteins. The phylogenetic tree was constructed for the (A) GLYI and (B) GLYII proteins from *Sorghum*, rice, *Arabidopsis*, *Medicago*, tomato, *Brassica*, soybean, and other plants using the Neighbour-Joining method in MEGA X with 1000 bootstrap replicates. Different clades indicated the different subgroups based on co-factor dependency and functional similarity.

Phylogenetic analysis was used to examine the sequence similarities and evolutionary divergence of GLYI and GLYII enzymes of tomato along with various other reported species, including *Arabidopsis*, rice, *G*. *max*, *Medicago*, *Sorghum*, and *Brassica*. The GLYI proteins were found to be separated and clustered into three major clades (Fig 2A). Clades I and II represented GLYI enzymes with Ni^2+^ and Zn^2+^ dependency, respectively. In contrast, clade III is formed by inactive/functional diverge GLYIs. Clade-I comprised of Ni^2+^-dependent rice GLYI proteins, including OsGLYI-2, OsGLYI-7, and OsGLYI-11 and *Arabidopsis* AtGLYI-3 and AtGLYI-6 [53, 57], suggesting Ni^2+^-dependent GLYI activity for members of SlGLYI in clade-I. Similarly, OsGLYI-8 and AtGLYI-2 in clade-II had Zn^2+^-dependent GLYI activity, indicating that other members of clade-II could be Zn^2+^-dependent enzymes [54]. Therefore, based on the members in these clades, the Ni^2+^-dependent GLYI isoform may be more prevalent in plants than the Zn^2+^-dependent isoform (Fig 2A).

Catalytic activity and metal dependency could be inferred for SlGLYI proteins by aligning their glyoxalase domain (PF00903) with Ni^2+^-dependent OsGLYI-11.2 and Zn^2+^-dependent OsGLYI-8 orthologues [53, 57], identifying the conserved actives site residues (S2A Fig). Conserved residues H/QEH/QE, present in both OsGLYI-8 and OsGLYI-11.2, defined the metal-binding site of GLYI enzymes, where glutamate residues receive proton from the substrate [53, 58]. Only four members, namely SlGLYI-2, SlGLYI-3, SlGLYI-6A, and SlGLYI-6B possessed all the conserved residues in their N terminal glyoxalase domain and could predicted as active GLYI members (Table 2). According to previous studies, proteins with two GLYI domains of approximately 120 amino acids each were the putative Ni^2+^-dependent forms, whereas those with a single GLYI domain of approximately 142 amino acids and two additional sequence stretches were considered the putative Zn^2+^-dependent [53, 57]. Based on this, SlGLYI-2 could be Zn^2+^ dependent, while the other three could be regarded as Ni^2+^ dependent.

**Table 2:** Information on domain organization of SlGLYI proteins for the prediction of enzymatic activity and metal ion dependency.

GLYI Proteins	GLYI domain			Presence of conserved metal-binding site				Expected enzyme activity	Metal ion dependency
	Start	End	length	H/Q	E	H/Q	E		
SlGLYI-1	11	131	121	-	-	-	-	No	-
SlGLYI-2	27	171	145	+	+	+	+	Active	Zn^2+^
SlGLYI-3	24	145	122	+	+	+	-	Active	Ni^2+^
	155	279	125	+	+	+	+		
SlGLYI-4A	14	133	120	+	-	+	+	No	-
SlGLYI-4B	18	137	120	+	-	+	+	No	-
SlGLYI-6A	148	255	108	+	+	+	-	Active	Ni^2+^
	265	385	121	-	+	+	+		
SlGLYI-6B	98	219	122	+	+	+	-	Active	Ni^2+^
	229	349	121	+	+	+	+		
SlGLYI-7A	13	132	120	+	-	+	+	No	-
SlGLYI-7B	9	128	120	+	-	+	+	No	-
SlGLYI-7C	5	122	118	+	-	+	+	No	-
SlGLYI-8	77	200	124	+	-	+	+	No	-
SlGLYI-9	24	151	128	+	-	+	+	No	-
SlGLYI-11	79	195	117	+	-	+	+	No	-

Similarly, GLYII proteins in the phylogenetic tree include OsGLYII-1 and AtGLYII-3 along with MtGLYII-12, GmGLYII-1, GmGLYII-2, GmGLYII-3 and GmGLYII-8 in clade-I, which were previously identified to have sulfur dioxygenase (SDO) activity [14, 59, 60], indicating that members of the GLYII family functionally diverged. The remaining clade consisted of the Glyoxalase II-like proteins (Fig 2B). All SlGLYII were regarded as functionally active as they had both active site (C/GHT) and metal ion binding site THHHXDH (Table 3), similar to that of AtGLYII-2 and OsGLYII-2, denoted with black lines in the multiple sequence alignment (S2B Fig).

**Table 3:** Information on domain organisation of SlGLYII proteins for the prediction of enzymatic activity.

Protein	Protein domain						Conserved metal binding / active site		Expected enzyme activity
	Metallo-beta-lactamase superfamily			Hydroxyacylglutathione hydrolase C-term					
	Start	End	length	Start	End	length	THHHXDH	C/GHT	
SlGLYII-1A	85	244	160	245	329	85	+	+	Active
SlGLYII-1B	229	391	163	-	-	-	+	+	Active
SlGLYII-3A	50	222	173	-	-	-	+	+	Active
SlGLYII-3B	51	223	173	-	-	-	+	+	Active

### Phylogenetic relationship, multiple sequence alignment and structural features of tomato DJ-1 members

**Fig 3:**
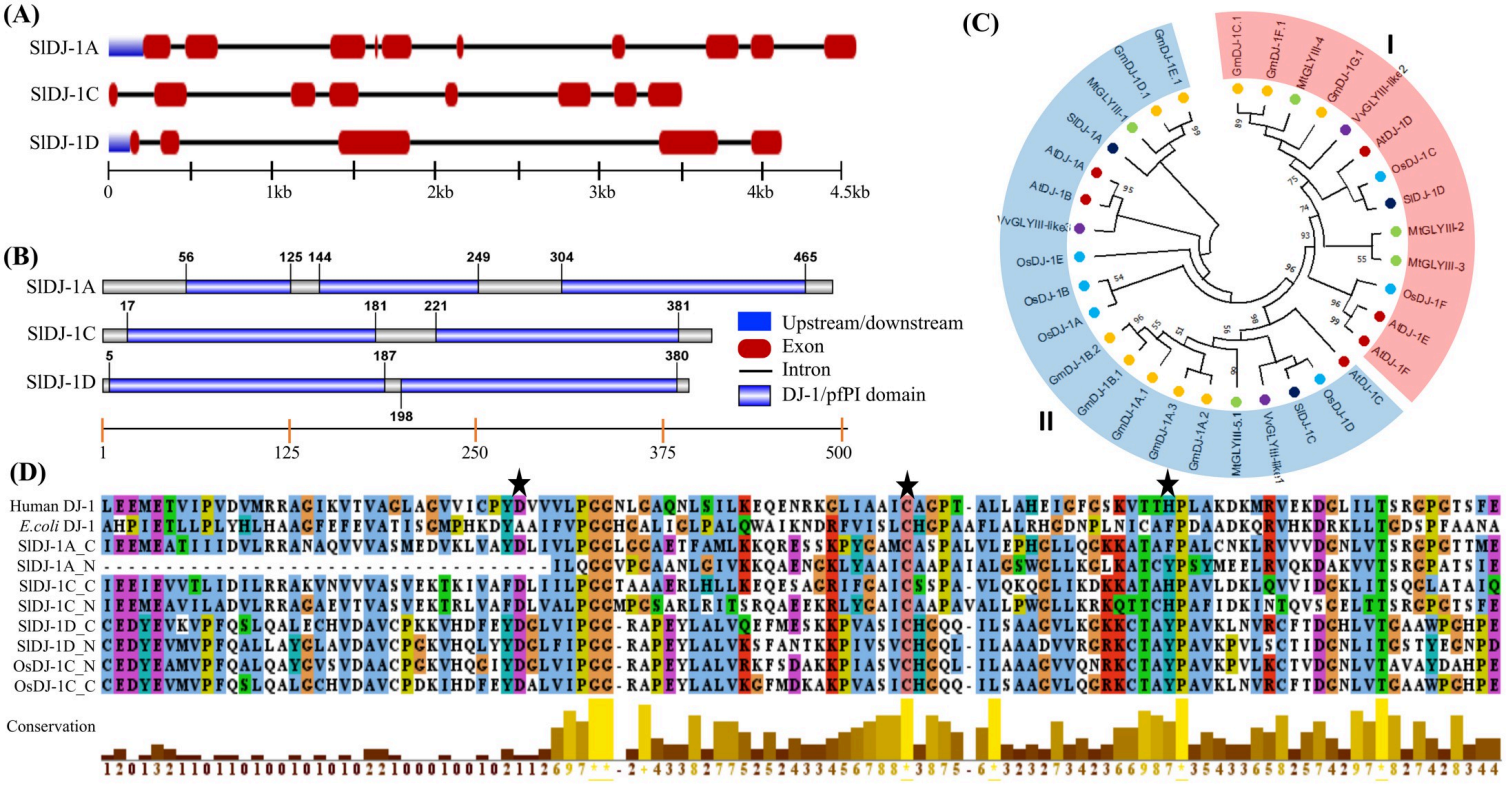
Phylogenetic relationship, sequence alignment, gene structure and domain architecture determination of SlDJ-1 members. (A) Gene structure was drawn by GSDS 2.0. (B) Schematic representation of domain organization of SlDJ-1 proteins. (C) A phylogenetic tree was built for the evolutionary conservancy among the DJ-1 members from various plant species by MEGA-X using the Maximum likelihood method with 1000 bootstrap. (D) Multiple sequence alignment was marked with black stars to indicate the conserved active site residues.

GSDS 2.0 was used to analyze the gene features of all identified DJ-1. SlDJ-1A possessed the most CDS regions, roughly ten, with a single upstream/downstream sequence. About five CDS regions were identified in the *SlDJ-1D* gene, compared to *SlDJ-1C*, which had eight. SlDJ-1A was the longest gene, whereas *SlDJ-1C* had the smallest (Fig 3A). In previous studies, DJ-1 proteins of plants such as *Arabidopsis* and rice were reported to have two DJ-1/PfpI domains, whereas humans, *Drosophila*, and *E*. *coli* only had a single domain [9]. The DJ-1/PfpI domain contained roughly 140 to 150 amino acids in most organisms, with the only exception being *E*. *coli*. According to multiple sequence alignment, most SlDJ-1 proteins had two consecutive DJ-1domains (Fig 3B) except for SlDJ-1A, which contained three DJ-1/PfpI domains of 70, 105, or 165 amino acids, respectively.

The phylogenetic relationship between DJ-1 proteins of different plant species illustrated the presence of two distinct groups (Fig 3C). Clade I indicated the active DJ-1 proteins, comprised of AtDJ-1D and OsDJ-1C, which were previously characterized to have GLYIII enzymatic activity [8, 9]. By contrast, clade II may contain DJ-1-like proteins which are partially active (Fig 3C). All SlDJ-1 proteins’ N-terminal DJ-1/PfpI domains were aligned with its orthologue in humans, bacteria and monocotyledon rice (OsDJ-1C). Three conserved residues, aspartate/glutamate, cysteine, and tyrosine/histidine, were found in all DJ-1 proteins [9] and denoted with black stars (Fig 3D). The core cysteine residue mainly contributes to the catalysis and, thus crucial for GLYIII enzymatic activity [61], which was found in all SlDJ-1 proteins (S3 Table). A previous study reported that *Arabidopsis* DJ-1 proteins, AtDJ-1e and AtDJ-1f, lacked either Asp/Glu or Tyr/His conserved residues, resulting in a partial loss of GLY III activity [8]. The most efficient GLYIII enzyme for tomato should be SlDJ-1A, which has all conserved amino acids, followed by SlDJ-1C.

### Phylogenetic relationship, multiple sequence alignment and structural features of tomato D-LDH

**Fig 4:**
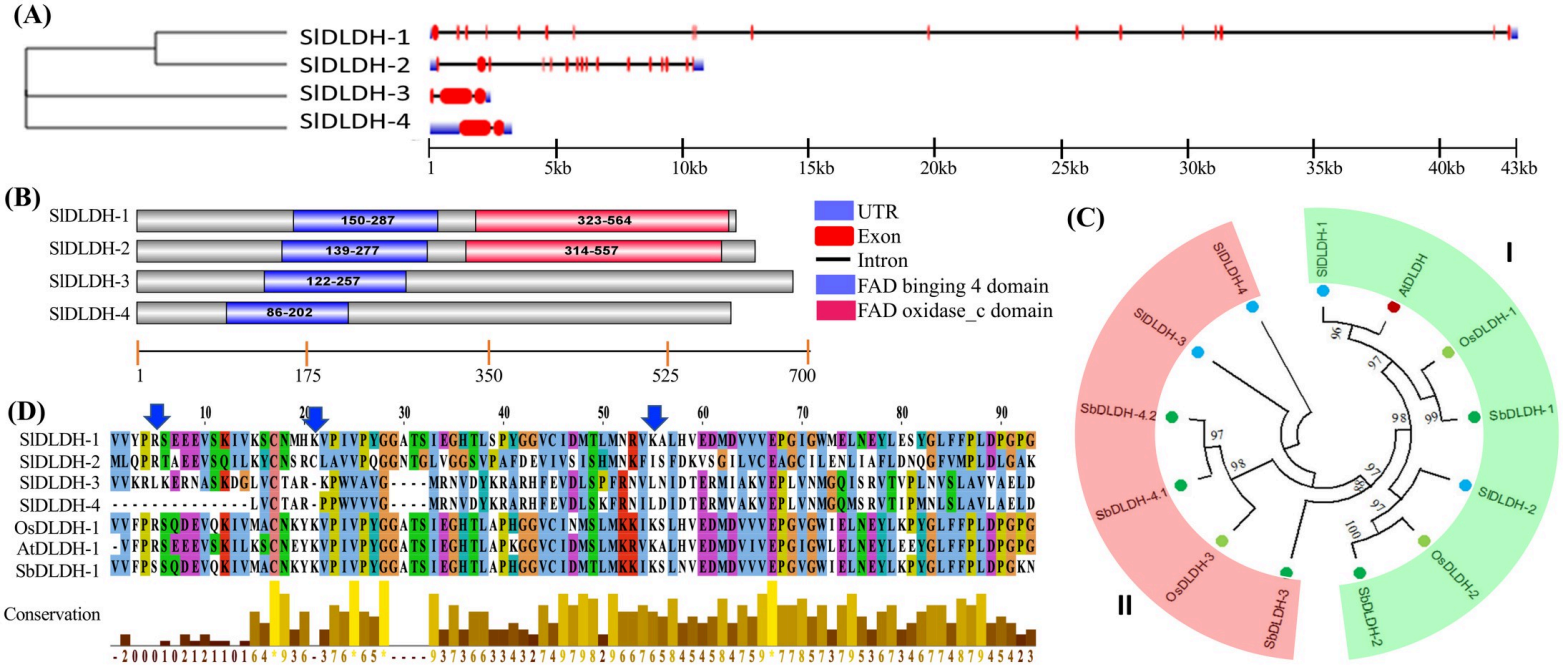
Elucidation of the structure and phylogenetic relationship DLDH members. (A) The exon-intron structure of *SlDLDH* genes. (B) Schematic representation of the domain architecture of SlDLDH proteins. (C) The evolutionary relationship of SlDLDH proteins with their orthologue from Sorghum, rice and *Arabidopsis* was observed by constructing a phylogenetic tree using MEGA-X with the Maximum Likelihood method and 1000 bootstrap. (D) Sequence conservance of SlDLDH proteins to identify the evolutionary conserved active residues that were marked with downward arrows.

The exon-intron patterns in identified SlDLDH genes were entirely distinct from one another (Fig 4A). *SlDLDH-1* possessed the greatest number of introns and exons, whereas *SlDLDH-2* possessed the fewest. Both of these proteins had a FAD-oxidase _C domain and a FAD_binding_4 domain. On the other hand, the remaining SlDLDH-3 and SlDLDH-4 proteins comprised the FAD_binding_4 domain solely (Fig 4B). Furthermore, phylogenetic analysis revealed that SlDLDH-1 and SlDLDH-2 cluster with AtDLDH, indicating that they are functionally comparable and likely to be mitochondrial proteins (Fig 4C). SbDLDH-3 and SlDLDH-4 proteins were more comparable to rice OsDLDH in terms of sequence similarities. All probable D-LDH proteins from tomato were analyzed with that of rice and *Arabidopsis* by sequence alignment (Fig 4D). The existence of highly conserved catalytic domains, denoted by black stars, suggested that the putative proteins are active (S4 Table).

### Structural insight of representative glyoxalase and D-LDH members

**Fig 5:**
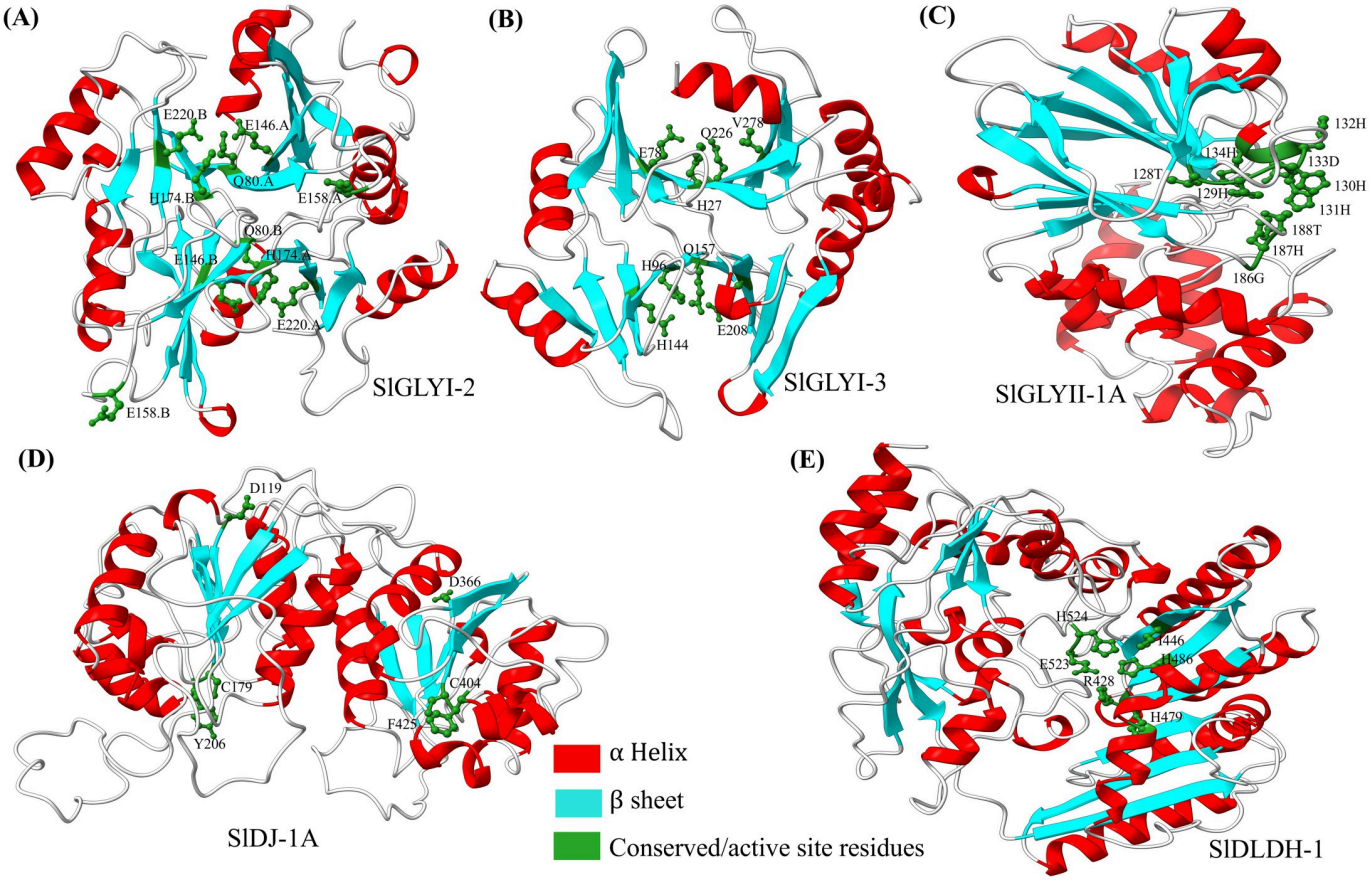
Three-dimensional structures of tomato glyoxalase and D-LDH proteins. Structures of SlGLYI-2 (A), SlGLYI-3 (B), SlGLYII-1A (C), SlDJ-1A (D), and SlDLDH-1 (E) were constructed using SWISS-MODEL, based on similar structures from the Protein Data Bank: *Gossypium hirsutum* GLYI (7VQ6), *Zea mays* GLYI (5D7Z), *Arabidopsis* AtGLYII-3 (2Q42), *Arabidopsis* AtDJ-1d (4OFW), and mouse mDLDH (8JDE), respectively. α-Helices are highlighted in bright red, β-sheets in cyan, and active site residues were visualized in a ball-stick model in forest green. Structures and active residues were visualized and annotated using ChimeraX.

The three-dimensional structures of SlGLYI-2, SlGLYI-3, SlGLYII-1A, SlDJ-1A, and SlDLDH-1 were predicted to understand the conformation of putative functional members (Fig 5). Zn^2+^-dependent SlGLYI-2 was modelled on *Gossypium hirsutum* glyoxalase (PDB: 7VQ6), revealing all the conserved residues Q80, E146, E158, H174, and E220 in the predicted homodimeric structure (Fig 5A), indicating functional activity. The Ni^2+^-dependent SlGLYI-3 was modelled after *Zea mays* glyoxalase I, and featured two glyoxalase domains with conserved residues H27, E78, H96, and E144 in N-terminal and Q157, E208, Q226, V278 in C terminal domain (Fig 5B). Notably C terminal glyoxalase featured a valine instead of conserved glutamate, indicating an inactive secondary actives site, similar to OsGLYI-11.2 [53]. The three-dimensional structure was SlGLYII-1A, based on *Arabidopsis* At2g31350 (PDB: 2Q42), featured all the residue in metal binding sites (128T, 129H, 130H, 131H, 132H, 133D, 134H) and active sites (186G, 187H, 188T), respectively (Fig 5C). The predicted model of SlDJ-1A, based on *Arabidopsis thaliana* DJ-1d (PDB: 4OFW) contained two DJ-1domain, featuring catalytic active residues D119, C179, Y206 for N- and D366, C404, F425 C-terminal domain (Fig 5D). While both of these domains contained the core cysteine residue, C-terminal DJ-1 lacked conserved Tyrosine. Further, SlDlDH-1 was modelled after the 8JDE, a recently solved crystal structure of mouse mitochondrial mDLDH [62], to shed light on the catalytic residues. In the predicted SlDLDH-1 structure, the active site residues were located at R428, I446, H479, H486, E523, and H524 (Fig 5E), which supposedly interact with Mn^2+^, FAD and Substrate D-lactate.

### Expression pattern analysis of glyoxalases and D-LDH in different developmental and anatomical stages

**Fig 6:**
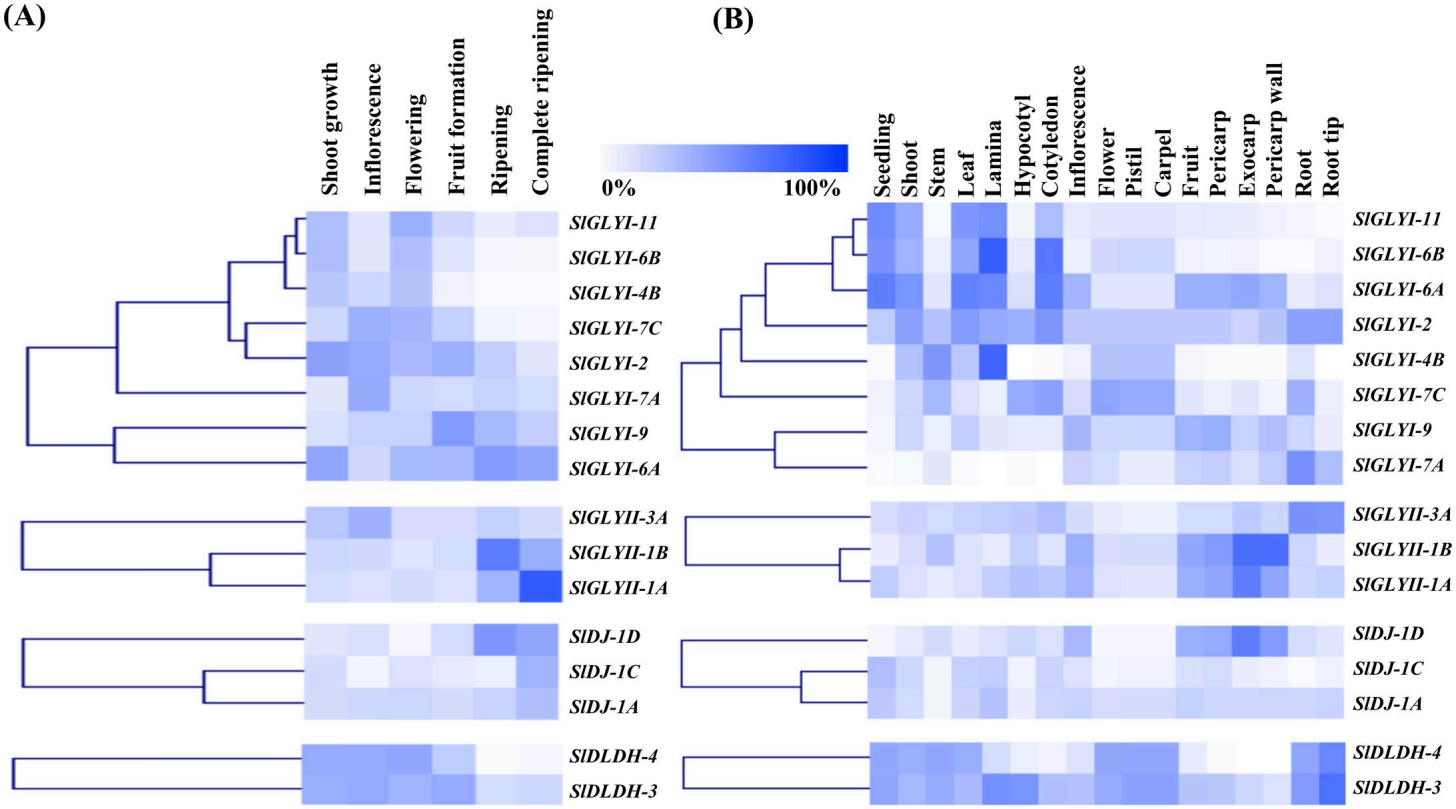
Expression profiling of methylglyoxal detoxifying genes from tomato. Expression profiling of glyoxalases and *DLDH* genes of tomato was performed with hierarchical clustering at (A) different developmental stages and (B) different anatomical tissues. Expression data were obtained for 17 different tissues and 6 distinct developmental stages from the Genevestigator database. The MeV software package (http://mev.tm4.org/) was used to build a heatmap with hierarchical clustering based on the Manhattan correlation. The level of expression can be interpreted by the colour scale where the intensity of colour is directly proportional to the level of expression.

Putative gene expression patterns of SlGLYI, SlGLYII, SlDJ-1, and D-LDH genes were obtained from a publically accessible microarray database using Genevestigator software to understand better the role of glyoxalases and D-LDH at distinct developmental stages and anatomical tissues (Fig 6). Expression analysis indicated that different tissues continuously expressed SlGLYI genes at varying levels. However, Ni^2+^-dependent *SlGLYI-6A’s* expression level was greater from seedling to flowering and shoot development stages (Fig 6A), opposite of inflorescence stages. By contrast, the expression of putative Zn^2+^-dependent *SlGLYI-2* was significantly increased in some developmental phases, except ripening and finished ripening. While all *SlDJ-1* expressed abundantly at the ripening stage, all *SlD-LDH* expressed oppositely. The expression levels of *SlGLYI-7A*, *SlGLYI-7C* and Zn^2+^-dependent *SlGLYI-2* were higher in underground tissue such as root and root tips. In contrast, Ni^2+^-dependent *SlGLYI-6A* and *SlGLYI-6B* showed greater expression in aerial tissue such as seedlings, shoots, leaves and lamina and cotyledon of the seed, while *SlGLYI-2* showed a similar expression pattern in these tissues (Fig 6B). Among *SlGLYII* genes, only *SlGLYII-3A* was expressed greatly in root and root tips, while *SlGLYII-1B* and *SlGLYII-1A* showed elevated expression in pericarp and exocarp tissue of fruits, which was similar to the case of *SlDJ-1D*. Both *SlDLDH-3* and *SlDLDH-4* were highly expressed in underground tissue like root and root tips, aerial tissue like shoot, steams and leaves as well as pistil and carpel tissue of flower.

### Expression profiling under different abiotic stress and pathogenic infections

**Fig 7:**
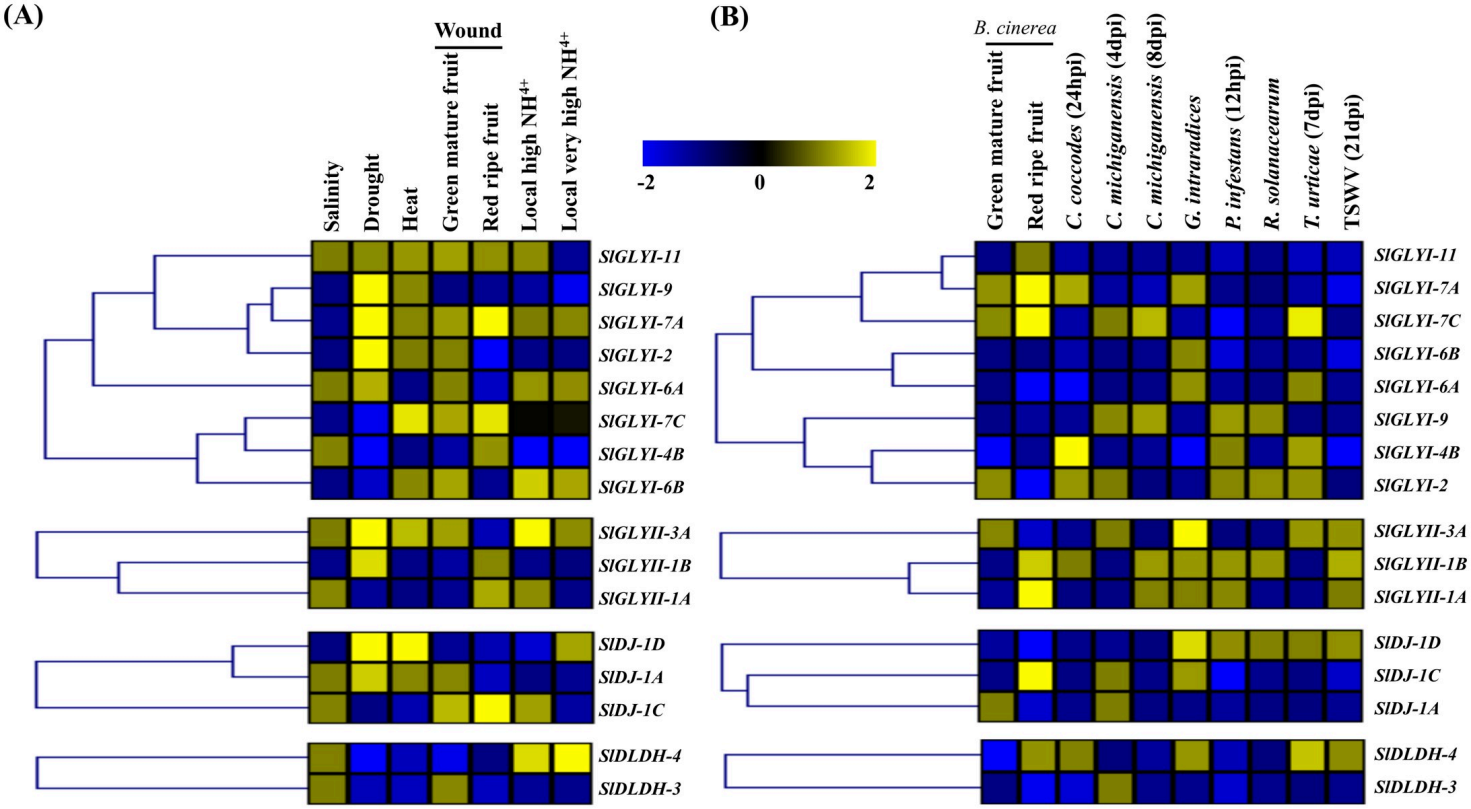
Expression analyses of tomato glyoxalases and *DLDH* genes in response to various abiotic and biotic stresses. Relative expression data of all the available *SlGLYI*s, *SlGLYII*s, *SlDJ-1*s, and *SlDLDH*s were retrieved from the genevestigator and combined and analyzed in response to different types and duration of (A) abiotic and (B) biotic stresses. Multiple Experiment viewer software packages (http://mev.tm4.org/) were used to construct the heatmaps with hierarchical clustering by using the Manhattan distance metric. The yellow squares in the heatmap indicate up-regulation and green indicates down-regulation of the corresponding transcripts.

The expression of all the identified *SlGLYI*, *SlGLYII*, *SlDJ-1* and *SlDLDH* transcripts were also analyzed under various pathogenic infections and abiotic stresses to understand their function in stress modulation of tomato through MG detoxification. The transcripts were expressed differentially in response to various abiotic stresses (Fig 7A). *SlGLYI*-9, *SlGLYI*-2, and *SlGLYI*-7A were upregulated in response to drought stress but were downregulated when salinity was applied. *SlGLYI*-9 expression was found to decrease in wounded fruits in either mature or ripening stages and when subjected to ammonium stress. Similarly, *SlGLYII*-3A and *SlGLYII*-B were upregulated during drought stress, and the expression somewhat decreased when exposed to salinity (Fig 7A). Among the analyzed SIGLYI, only *SIGLYI*-4B and *SIGLYI*-6A expressed at a reduced level in response to heat, and all SIGLYII except *SIGLYII*-3A expressed at a similar pattern. When exposed to heat, *SIDJ-1D* and *SIDJ-1A* were found to be upregulated. In response to local high and very high concentrations of ammonium, the transcript abundance of *SlGLYII*-*6B*, *SlGLYII*-3A, and *SlDLDH*-4 was highly expressed as compared to the other analyzed genes. Overall, most of the glyoxalase system enzymes were expressed at the highest level in response to drought stress, followed by heat and salinity. Most glyoxalase genes were upregulated in drought stress, while D-LDH were mostly upregulated in salinity and ammonium stress.

When tomato seedlings were subjected to a variety of biotic stresses, the *SlGLYI* gene expression pattern altered significantly (Fig 7B). *SlGLYI-7A* and *SlGLYI-7C* were highly upregulated in response to pathogen *B*. *cinerea*. However, their expression decreased when exposed to other pathogens. SlGLYII enzymes may play a larger part in the defence system since they are mostly upregulated in response to various pathogens, especially *SlGLYII*-1A and *SlGLYII*-1B. Among *SlDJ-1* genes, only SIDJ-1D was elevated in tomato seedlings following infection with *C*. *michiganensis*, *C*. *coccodes*, *G*. *intraradices*, *P*. *infestans*, *R*. *solanacearum*, and *T*. *urticae* strains. Several *SlGLYI* and *SlDLDH* genes were more highly expressed, including *SlGLYI-9*, *SlGLYI-2*, *SlGLYI-4B*, and *SlDLDH-4*, whilst others exhibited little or no transcript alteration.

### Validation of selected SlGLY, SlDJ-1, and SlDLDH genes under various abiotic stresses

**Fig 8:**
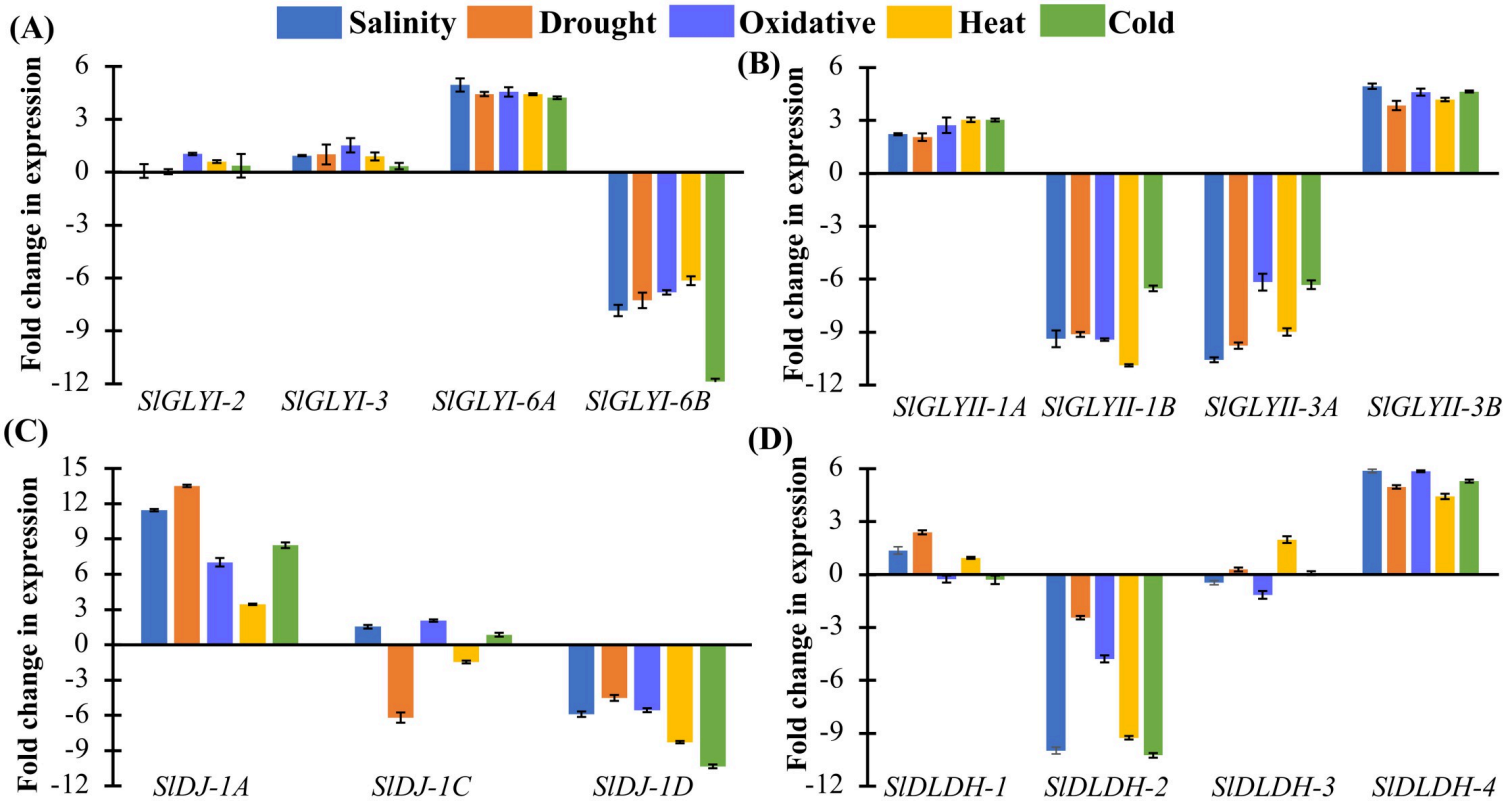
Abiotic stress-responsiveness of putative functional MG detoxifying members in tomato by qRT-PCR. The expression pattern of in silico analysed putative functional four *SlGLYI* (A), four *SlGLYII* (B), three *SlDJ-1* (C), and four *SlDLDH* (D) genes was investigated in 10d old tomato seedlings by exposing different abiotic stress including salinity, drought, oxidative, heat, and cold stress for 6 h. Bar diagrams illustrate the average fold changes in gene expression relative to controls using *SlEF1α* as a reference gene. Each bar is distinctly coloured based on the type of stress conditions.

The abiotic stress‑responsiveness expression of the predicted enzymatically functional glyoxalase and *D-DLDH* genes were further studied in tomato using quantitative RT-PCR analysis under various stress conditions, including salinity, drought, oxidative, heat, and cold stresses (Fig 8 and S5 Table). All the predicted Ni^2+^-dependent *SlGLYI* genes namely, *SlGLYI-3*, *SlGLYI-6A*, and *SlGLYI-6B* were found to be more stress-inducible as compared with the only Zn^2+^-dependent *SlGLYI-2* to all the stress treatments (Fig 8A). The putative Zn^2+^-dependent *SlGLYI-2* showed marginal fluctuation in expression in all the stresses except for oxidative stress. Interestingly, three Ni^2+^-dependent members *SlGLYI-3*, *SlGLYI-6A*, and *SlGLYI-6B* behaved differently where *SlGLYI-3* showed slight upregulation in expression in all the conditions, *SlGLYI-6A* showed more than 4 times upregulation, and *SlGLYI-6B* exhibited more than 6 folds downregulation.

Similarly, four functionally active GLYII genes namely *SlGLYII-1A*, *SlGLYII-1B*, *SlGLYII-3A*, and *SlGLYI-3B* also showed stress-mediated perturbations differently. Among them, two members such as *SlGLYII-1A*, and *SlGLYI-3B* showed more than 2 folds in expression in all five stress conditions, while the other two members *SlGLYII-1B* and *SlGLYII-3A* showed more than 6 folds of downregulation in expression (Fig 8B).

Similarly, the expression of three noble GLYIII-like genes *SlDJ-1A*, *SlDJ-1B*, and *SlDJ-1C* was analyzed in tomato. *SlDJ-1A* showed significant upregulation under all these stress conditions, while *SlDJ-1C* showed significant downregulation (Fig 8C). The other member, *SlDJ-1B* showed a mixed pattern of expression where salinity, oxidative and cold stresses brought slight upregulation and drought and heat stresses induced downregulation of the gene (Fig 8C).

Finally, expression profiling of all four identified SlDLDH isoforms was performed under five abiotic stress conditions (Fig 8D). The alteration in the expression of *SlDLDH-1* and *SlDLDH-3* was marginal as compared to the other two genes. *SlDLDH-2* showed downregulation in the expression of more than 4 folds, while the expression of *SlDLDH-4* was more than 4 folds upregulated under all stress conditions, potentially reflecting its role in metabolizing D-lactate during stress conditions.

### Identification of putative cis-regulatory elements in the promoter region

**Fig 9:**
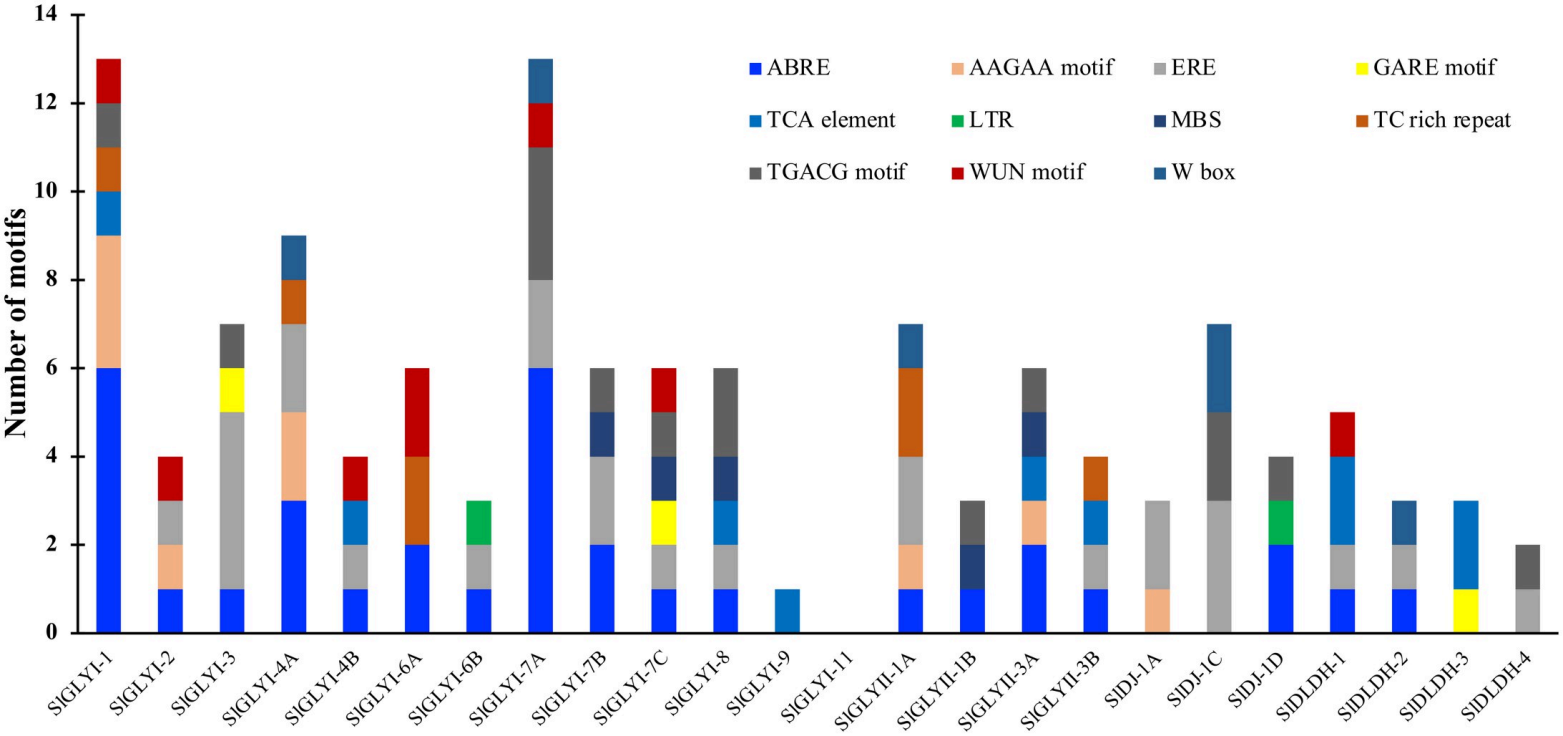
In silico promoter analysis of SlGLYI, SlGLYII, SlDJ-1 and SlDLDH members. The 1 kb upstream sequence of all the candidate genes was retrieved from the phytozome database and scanned through PlantCARE. The core promoter elements such as TATA-box and CAAT-box were not shown in the figure for better visibility. The number of stress-related and hormonal-related -cis-elements was determined for the analyzed promoter sequences.

Cis-regulatory elements were identified in the promoter region of the glyoxalases and D-LDH genes to understand their role in transcriptional regulation during developmental and environmental stimuli (Fig 9). The 1000 bp upstream region of *SlGLY* and *SlDLDH* transcripts were retrieved from the phytozome database and scanned to identify cis-elements in the promoter. Various hormone-responsive elements in the identified genes included abscisic acid-responsive (ABRE), ethylene-responsive (ERE), gibberellin-responsive (GARE), salicylic acid-responsive elements (TCA-element), and MeJA-responsive element (TGACG-motif) were identified in the study. Cis-elements associated with defence and stress response were also discovered, including fungal elicitor-responsive (W-Box), and low-temperature-responsive (LTR elements) (S3 Fig). Except for *SlGLYI-11*, which had no stress-related motif in its upstream sequence, most SlGLY and SlDLDH members had at least one hormone-responsive and one stress-responsive element in their putative promoter region. The ABRE, ERE, and TGCAG-motif were the top three most prevalent elements implicated in hormone and stress responses. The promoters of *SlGLYI-1* and *SlGLYI-7* have the most cis-elements 13, followed by *SlGLYI-4B* with 7 (Fig 9 and S6 Table). The positive alteration of the SlGLY and SlDLDH expression and their enzymatic activity under various abiotic stress conditions could be directly correlated with the presence of such a broad array of hormones and stress-inducible cis-elements.

### Effect of various abiotic stress on the total activity of GLYI and GLYII enzymes

**Fig 10:**
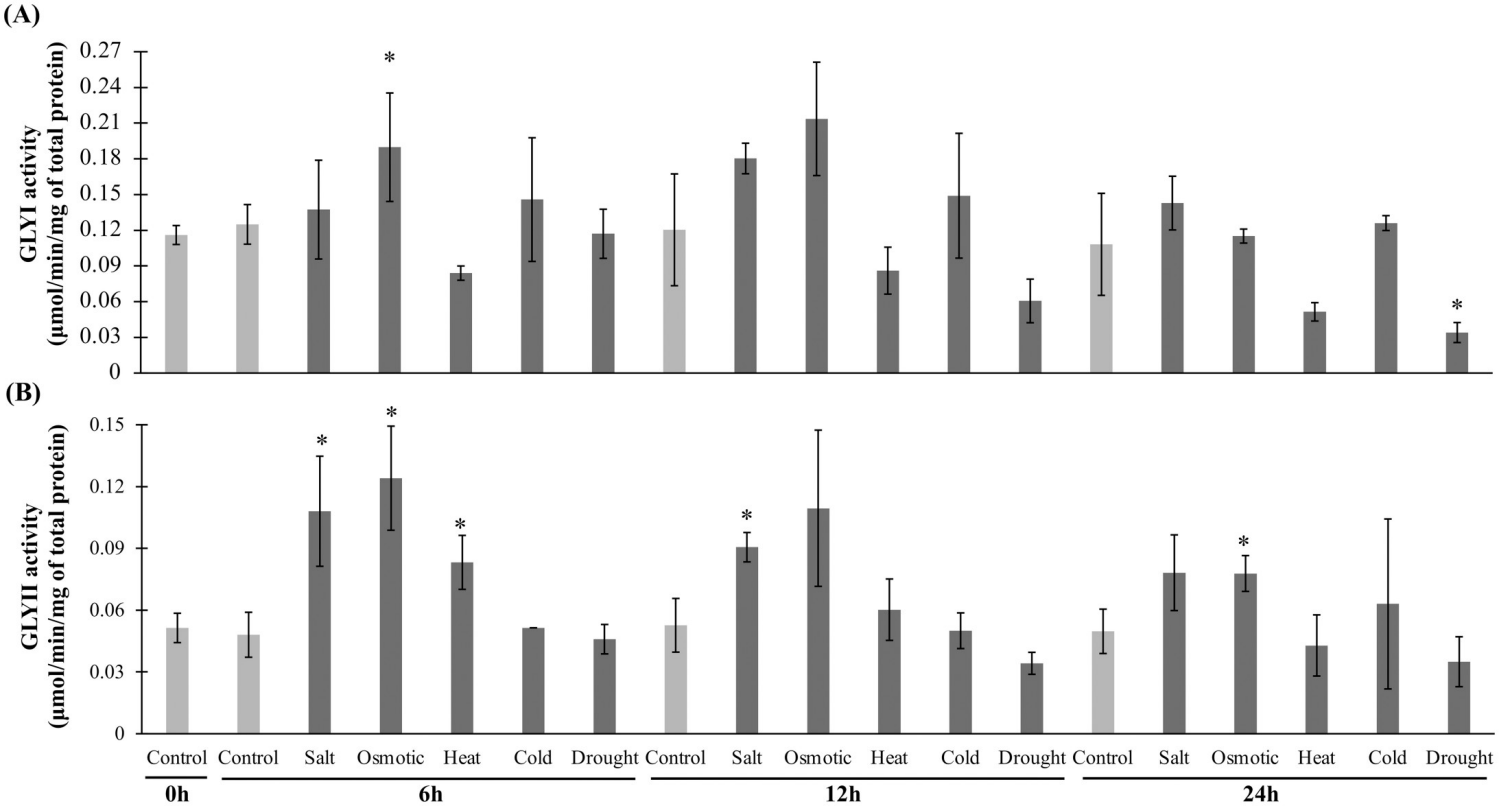
Measurement of total GLYI and GLYII activity in response to various abiotic stresses. Total GLYI (A) and GLYII (B) enzyme activity were measured in response to various abiotic stresses such as salinity, osmotic, heat, cold, heat, and drought stresses at four different time points of stress exposure (0h to 24h). The activity was represented as μmoles/min/mg of total protein. All the experiments were repeated thrice and represented as the average ± standard deviation (n = 3).

To gain deep insights into the function of glyoxalase genes in the abiotic stress adaptation in tomato, the total GLYI and GLYII activity was measured in response to various abiotic stress conditions including salinity, osmotic stress, heat, cold, heat, and drought, and compared with the respective untreated control conditions (Fig 10 and S7 Table). The plants were grown normally and exposed to different stresses in the laboratory. The activity was measured in four different time points such as 0h, 6h, 12h and 24h and expressed as μmol/min/mg of total protein. An enhancement of total GLYI activity was observed for salinity and osmotic stress. However, the activity decreased over time (Fig 10A). On the other hand, heat, cold and drought negatively affected the total GLYI activity, which gradually decreased over time. A similar pattern was observed for the total GLYII activity where salinity, osmotic and heat (to some extent) enhanced the activity (Fig 10B). Both the activity of GLYI and GLYII enzymes were found to be gradually decreasing over time, indicating that inverse relation with the extent of stress exposure.

## Discussion

Climate change, which results in various forms of abiotic stresses, is the main contributor to yield losses in various economically significant crops worldwide. Crops prone to dryness, high temperatures, extreme cold, and saline soil as a result of global warming suffer considerable yield losses. The production of tomato has continuously declined in the last century due to adverse climatic conditions. The metabolic byproduct MG is produced spontaneously in all living organisms [6], while the level goes up in response to diverse abiotic stressors [20]. The importance of the glyoxalase system in eliminating MG and providing tolerance from a variety of abiotic stressors has already been established through previous research [5, 6]. Previous studies had also identified glyoxalase genes in different plant species, including *Arabidopsis* (10 GLYI, 5 GLYII, and 6 DJ-1) (8,26), Soybean (24 GLYI, 12 GLYII, and 7 DJ-1) [14], *M*. *truncatula* (29 GLYI, 14 GLYII, and 5 DJ-1) [60] and rice (11 GLYI, 3 GLYII, and 6 DJ-1) [26]. Four D-LDH genes were reported in *Sorghum bicolor*, with 15 GLYI and 6 GLYII genes [49]. This study identified 13 putative GLYI, 4 GLYII, 3 DJ-1, and 4 DLDH genes in the tomato genome *Solanum lycopersicum ITAG3*.*2* (Table 1).

Among the identified members in tomato, three duplication events were observed in this study (Fig 1B). Two duplication events were discovered among the conventional glyoxalase genes (GLYI and GLYII), whereas one duplication was found in the SlDLDH genes. All duplication events were determined to be segmental (S2 Table). The *SlGLYI-4A/7A* pair was formed 37.9 Mya ago, whereas *SlGLYII-3A/3B* and *SlDLDH-3/4* were formed 23.6 and 29.5 Mya ago, respectively. In contrast, soybean exhibits ten duplication patterns among glyoxalase genes (GLYI and GLYII) [14], whereas five GLYI and one GLYII gene have reported such occurrences in *Medicago* [60]. These duplication events in plants have led to gene gain/loss and confer abiotic stress modulation [63].

The predicted SlGLYI proteins may be classified into two categories based on their activity, similar to their orthologues in other plant species. Active GLYI is either Zi^2+^- or Ni^2+^-dependent. The metal selectivity of SlGLYI proteins was estimated based on their domain sequencing and length, as indicated in the previous studies [57]. Only four GLYI proteins, SlGLYI-2, SlGLYI-3, SlGLYI-6A and SlGLYI-6B, were considered active as they had conserved metal-binding sites (Table 2). SlGLYI-2 was discovered as a Zn^2+^-dependent glyoxalase protein and had a domain length of 143 amino acids (S1B Fig), whereas the remaining three proteins are likely to be Ni^2+^-dependent with domain lengths of approximately 120 amino acids, quite similar to its orthologues in rice [53, 57]. Other identified SlGLYI might be functionally inactive GLY-like proteins that lacked the conserved motif.

SlGLYII-3A and SlGLYII-3B were discovered as functionally active GLYII enzymes due to their conserved metal-binding site THHHXDH (Table 3) and high sequence similarity to rice’s functionally active OsGLYI-2 and OsGLYI-3 proteins [20]. SlGLYII-1A and SlGLYII-1B, on the other hand, were potential SDO enzymes based on sequence similarity to the OsGLYII-2 and AtGLYII-2 proteins [59] that exhibited SDO activity, indicating functional divergence among GLYII enzymes (S2B Fig). In previous research, the Hydroxyacylglutathione hydrolase (HAGH_C) domain was also discovered in the C terminal of functionally active GLYII enzymes by sequencing and crystal structure analysis postulated that substrate binding might occur at the interface between HAGH_C and the catalytic β-lactamase region [56]. As a result, the original GLYII enzyme may have an additional HAGH_C domain, as confirmed with SlGLYII-1A (S2B Fig).

In-silico investigation of SlDJ-1 proteins found three candidates in tomato with DJ-1 domain (Table 1), whereas five members were identified in *Medicago* [60], six in *Arabidopsis* and rice [8, 26] and seven in soybean [14]. Previous research on DJ-1 protein revealed plants had three major types of DJ-1 proteins, diverged from prokaryotes, protists and green algae [64]. Most of the plant-derived DJ-1 proteins had double DJ-1/PfpI domain, except for date palm, which included up to four [65]. In the glutathione-independent pathway, the DJ-1/PfpI containing glyoxalase III enzyme converts MG to harmless D-lactate in a single-step reaction [9]. D-LDH enzymes belonging to the FAD_binding_4 superfamily catalyze D-lactate’s transformation to pyruvate. In the HMM profile search, 24 such members were initially identified as belonging to the FAD-binding superfamily. In addition to FAD binding 4, these identified members featured several additional domains, demonstrating the functional variety of the group. To anticipate SlDLDH members that specifically catalyze D-Lactate transformation, SlDLDH containing a single FAD_binding_4 or an additional FAD-oxidase_C domain were selected according to a prior study [49]. Therefore, four such enzymes with specific D-lactate catalytic activity were found in tomato, two of which had an additional FAD-oxidase C domain. Homology modelling of SlDLDH-1 revealed that it contained all the active site residues at R428, I446, H479, H486, E523, and H524 to catalyze D-lactate (Fig 5E), indicative of functional activity.

Previous research on rice and *Arabidopsis* identified glyoxalase genes to be highly selective to certain tissues or developmental stages [26]. In a similar study on *M*. *truncatula*, *MtGLYI-4* showed the greatest expression level across all 17 analyzed tissues, while *MtGLYI-3*, *MtGLYI-18*, and *MtGLYI-20* had the lowest expression levels [60]. In the current study, we used publicly available microarray data and observed a cluster of GLYI genes, including *SlGLYI-11*, *SlGLYI-6B*, and *SlGLYI-6A*, expressed in great abundance in aerial tissue like shoot, leaf and lamina while showing minimal expression in underground tissues such as root, root tips (Fig 6B). Recent research has shown that one of the rice glyoxalases, *OsGLYI-3*, promotes seed lifespan and salt tolerance in rice plants [66]. *SlGLYI-11* may serve a similar function as it showed significant expression in seed tissue and was highly elevated under saline conditions. However, further experimental evidence for functional characterization.

Tomato GLYII member *SlGLYII-3A* was significantly expressed in root and root tips while *SlGLYII-1B* and *SlGLYII-1A*showed the highest expression level in fruits’ Exocarp and Pericarp walls (Fig 6B). This demonstrates the presence of functional diversity among the glyoxalase II protein in various developmental cues; a similar phenomenon was observed in the case of soybean, where *GmGLYII-12*, *GmGLYII-5* and *GmGLYII-4* were found to be only expressed in seed tissue, while *GmGLYII-9* was expressed in seeds, roots and flower tissue [14]. *SlDJ-1D* showed the highest expression in fruit tissue while the expression of *SlDJ-1C* and *SlDJ-1A* was very minimal (Fig 6B). Similar phenomenon was observed in *M*. *truncatula* where *MtDJ-1D* and *MtDJ-1A* showed expression equally in underground, aerial and seed tissue, while *MtDJ-1C* and *MtDJ-1B* showed no expression [60]. D-lactate dehydrogenase in *Sorghum bicolor*, *SbDLDH-3* and *SbDLDH-4* showed a higher level of expression in root and shoot tissue [49], similar to *SlDLDH-3* in tomato, which is also highly expressed in the root (Fig 6B), indicating its common role of root development in different plant species. The increased expression of *SlGLYII-1B*, *SlGLYII-1A*, and *SlDJ-1D* genes during the fruit ripening stage revealed that GLYII and DJ-1 m play may play some roles in fruit maturation of tomato (Fig 6A). However, the majority of GLYI and D-LDH may play a part in shoot formation, Inflorescence and flower development, as can be observed from the heatmaps (Fig 6A).

In previous studies on rice [26], *G*. *max* [14] and *Medicago* [60], different members of the glyoxalase family had been identified as potential modulators of abiotic stresses. In this study, the transcriptomic data from Genevestigator revealed that most of the glyoxalase genes were upregulated in response to drought, followed by heat and salinity. In most scenarios, upregulated genes are clustered together, a pattern that is also common for downregulated genes (Fig 7A). Members of the *SlGLYI* gene family- *SlGLYI-9*, *SlGLYI-7A*, along with *SlGLYII-3A* and *SlDJ-1D* were elevated in response to dehydration and heat stress but decreased in response to salinity exposure. The Ni^2+^-dependent *SlGLYI-6A* exhibited increased expression under salinity and drought conditions, whereas *SlGLYI-6B* was downregulated. Conversely, the Zn^2+^-dependent *SlGLY-2* was primarily expressed in response to drought, heat, and physical stress (Fig 7A).

To validate the expression pattern of glyoxalase genes in tomato, we utilized quantitative RT-PCR to observe the expression of four representative GLYI, including *SlGLYI-2*, *SlGLYI-3*, *SlGLYI-6A*, and *SlGLYI-6B* under abiotic stress (Fig 8). Ni^2+^-dependent *SlGLYI-3* and *SlGLYI-6A* were upregulated in stress conditions, while *SlGLYI-6B* was highly downregulated. Zn^2+^-dependent *SlGLYI-2* also showed minimal upregulation against abiotic stressors (Fig 8A). In soybean, Ni^2+^-dependent *GmGLYI-1*, *GmGLYI-2*, and *GmGLYI-3* showed downregulation, while Zn^2+^-dependent *GmGLYI-15* and *GmGLYI-16* were upregulated [14]. In *Medicago*, most of the *MtGLYI* genes were upregulated in response to drought stress, while most MtGLYII remained unchanged in expression pattern. However, a cluster of *MtGLYI* and most of the *MtGLYII* were downregulated during salinity stress [60]. In contrast, a heterogeneous expression pattern of *SlGLYII* genes was observed where *SlGLYII-1B* and *SlGLYII-3A* were downregulated under stress conditions and *SlGLYII-1A* and *SlGLYII-3B* were upregulated (Fig 8B). These observations suggested a functional divergence among conventional glyoxalase family members across different plant species, highlighting their varied adaptations and responses to environmental stressors.

In previous research, the DJ-1 members of prokaryotes *Escherichia coli* and eukaryotes *S*. *cerevisiae* displayed salt tolerance [67], which may be confirmed by the upregulation of tomato *SlDJ-1A* and *SlDJ-1C* in response to salinity (Fig 8C). A similar phenomenon was observed in *Medicago*, in which all of the *MtDJ-1* members were expressed under salinity stress along with other stress conditions [60]. A recent investigation into tomato glyoxalase III (GLYIII) genes revealed that their overexpression enhances salt and osmotic stress tolerance, contributing positively to plant growth and yield [68]. Further, transgenic tobacco expressing *E*. *coli* DJ-1 demonstrated enhanced salt tolerance during vegetative and reproductive stress. Transgenic sugarcane overexpressing GLYIII (EaGlyIII) also improves salt and drought tolerance, according to a recent study [69]. Among SlDLDH members, *SlDLDH-4* was mostly upregulated under abiotic stress conditions while *SlDLDH-2* was highly downregulated. In contrast *SlDLDH-1* and *SlDLDH-3* showed heterogenous expression in response to abiotic stress (Fig 8D).

Moreover, microarray data revealed transcript abundance of *SlGLYI-7A* and *SlGLYI-7C* of the GLYI family, *SlGLYII-1B* and *SlGLYII-1A* of the GLYII family, along with *SlDJ-1C* and *SlDLDH-4* against the pathogenic fungal infection of *Botrytis cinerea*. Furthermore, most of the *SlGLYII*s with *SlGLYI-7A*, *SlGLYI-2*, *SlDJ-1D*, and *SlDLDH-4* showed greater responses against various pathogenic infections (Fig 7B). Alongside with qPCR data, the activity of total GLYI and GLYII enzymes was found to gradually decrease with the increment of stress duration, indicating the physiological response of tomato glyoxalase in stress modulation (Fig 10). Our study on the functional characterization of MG detoxifying genes could lead to the development of a stress-tolerant tomato variety with improved pathogen resistance.

## Conclusion

To summarise, genome-wide investigation of methylglyoxal detoxifying enzymes in tomato led us to identify 13 GLYI, 4 GLYII, 3 DJ-1, and 4 D-LDH genes distributed in 9 different chromosomes. Three segmental duplication events were observed. Based on the previous literature study, sequence analysis, the presence of conserved domain architecture, the metal ion dependency and putative enzymatic activity of the identified proteins have been predicted. The expression data confirmed the significant role of these genes in different developmental stages, anatomical tissues, and stress modulation pathways by mitigating MG accumulation during different biotic and abiotic stresses. *SlGLYI-6A*, *SlGLYII-3B*, *SlDJ-1A* and *SlDLDH-4* of the MG detoxifying gene family showed significant upregulation in response to environmental stressors, while *SlGLYII-1B*, *SlGLYII-1A*, *SlGLYI-2*, *SlDJ-1D*, *SlDLDH-4* were highly expressed in response pathogens, indicating their role in plant defense systems and stress modulation. Further functional analysis of these genes may help to develop an engineered stress-tolerant plant.

## Supporting information

S1 Table Primer sequences of selected *SlGLY* and *SlDLDH* genes were used in the study. (DOCX)

S2 Table Gene duplication event between tomato glyoxalases and D-lactate dehydrogenase genes. (DOCX)

S3 Table Information on domain organisation of SlDJ-1 proteins for the prediction of enzymatic activity. (DOCX)

S4 Table Information on domain organisation of SlDLDH proteins for the prediction of enzymatic activity. (DOCX)

S5 Table Raw data for quantitative RT-PCR analysis under various stress conditions, including salinity, drought, oxidative, heat, and cold stresses. (XLS)

S6 Table The presence of cis-regulatory elements in the promoter region in *SlGLY* and *SlDLDH* genes. (DOCX)

S7 Table Raw data for total GLYI and GLYII enzyme activity. (XLSX)

S1 Fig Structural feature of SlGLYI and SlGLYII genes and proteins. (TIF)

S2 Fig Sequence alignment and active site analysis of the conventional GLYI and GLYII enzymes. (TIF)

S3 Fig In silico analysis of SlGLYI, SlGLYII, SlDJ-1 and SlDLDH promoters. (TIF)

## Abbreviations

aa: amino acid
bp: base pair
D-LDH: D- lactate dehydrogenase
GSH: reduced glutathione
h: hour
MG: methylglyoxal
Mya: million years

## References

[1] Thornalley PJ. Pharmacology of methylglyoxal: Formation, modification of proteins and nucleic acids, and enzymatic detoxification—A role in pathogenesis and antiproliferative chemotherapy. Gen Pharmacol. 1996;27(4):565–73. doi: 10.1016/0306-3623(95)02054-3 8853285

[2] Thornalley PJ. Protein and nucleotide damage by glyoxal and methylglyoxal in physiological systems—role in ageing and disease. Drug Metabol Drug Interact [Internet]. 2008 [cited 2022 Jan 13];23(1–2):125–50. Available from: https://pubmed.ncbi.nlm.nih.gov/18533367/ doi: 10.1515/dmdi.2008.23.1-2.125 18533367 PMC2649415

[3] Thornalley PJ, Waris S, Fleming T, Santarius T, Larkin SJ, Winklhofer-Roob BM, et al. Imidazopurinones are markers of physiological genomic damage linked to DNA instability and glyoxalase 1-associated tumour multidrug resistance. Nucleic Acids Res [Internet]. 2010 Apr 30 [cited 2022 Jan 13];38(16):5432–42. Available from: https://pubmed.ncbi.nlm.nih.gov/20435681/ doi: 10.1093/nar/gkq306 20435681 PMC2938218

[4] Shivani Grewal SK, Gill RK, Virk HK Bhardwaj RD. Methylglyoxal detoxification pathway—Explored first time for imazethapyr tolerance in lentil (Lens culinaris L.). Plant Physiol Biochem. 2022 Apr 15;177:10–22. doi: 10.1016/j.plaphy.2022.02.007 35219898

[5] Kaur C, Ghosh A, Pareek A, Sopory SK, Singla-Pareek SL. Glyoxalases and stress tolerance in plants. Biochem Soc Trans. 2014;42(2):485–90. doi: 10.1042/BST20130242 24646265

[6] Kaur C, Singla-Pareek SL, Sopory SK. Glyoxalase and Methylglyoxal as Biomarkers for Plant Stress Tolerance. CRC Crit Rev Plant Sci. 2014;33(6):429–56.

[7] Lee JY, Song J, Kwon K, Jang S, Kim C, Baek K, et al. Human DJ-1 and its homologs are novel glyoxalases. Hum Mol Genet [Internet]. 2012 Jul [cited 2022 Jan 13];21(14):3215–25. Available from: https://pubmed.ncbi.nlm.nih.gov/22523093/ doi: 10.1093/hmg/dds155 22523093

[8] Kwon K, Choi D, Hyun JK, Jung HS, Baek K, Park C. Novel glyoxalases from Arabidopsis thaliana. FEBS J. 2013;280(14):3328–39. doi: 10.1111/febs.12321 23651081

[9] Ghosh A, Kushwaha HR, Hasan MR, Pareek A, Sopory SK, Singla-Pareek SL. Presence of unique glyoxalase III proteins in plants indicates the existence of shorter route for methylglyoxal detoxification. Sci Rep. 2016;6(March 2015):1–15. doi: 10.1038/srep18358 26732528 PMC4702089

[10] Jain M, Aggarwal S, Nagar P, Tiwari R, Mustafiz A. A D-lactate dehydrogenase from rice is involved in conferring tolerance to multiple abiotic stresses by maintaining cellular homeostasis. Sci Rep [Internet]. 2020;10(1):1–17. Available from: doi: 10.1038/s41598-020-69742-0 32732944 PMC7393112

[11] Urscher M, Przyborski JM, Imoto M, Deponte M. Distinct subcellular localization in the cytosol and apicoplast, unexpected dimerization and inhibition of Plasmodium falciparum glyoxalases. Mol Microbiol. 2010;76(1):92–103. doi: 10.1111/j.1365-2958.2010.07082.x 20149108

[12] Talesa V, Rosi G, Contenti S, Mangiabene C, Lupattelli M, Norton SJ, et al. Presence of glyoxalase II in mitochondria from spinach leaves: comparison with the enzyme from cytosol. Biochem Int. 1990 Dec;22(6):1115–20. 2090107

[13] Deswal R, Chakaravarty TN, Sopory SK. The glyoxalase system in higher plants: Regulation in growth and differentiation Establishment of the presence of the glyoxalase system in plants Presence of methylglyoxal in plant tissues and its effect Effect of exogenous factors on glyoxalase levels. 1989;(1979).

[14] Ghosh A, Islam T. Genome-wide analysis and expression profiling of glyoxalase gene families in soybean (Glycine max) indicate their development and abiotic stress specific response. BMC Plant Biol [Internet]. 2016;16(1):1–25. Available from: doi: 10.1186/s12870-016-0773-9 27083416 PMC4833937

[15] Aronsson AC, Marmstål E, Mannervik B. Glyoxalase I, a zinc metalloenzyme of mammals and yeast. Biochem Biophys Res Commun [Internet]. 1978 Apr 28 [cited 2022 Jan 14];81(4):1235–40. Available from: https://pubmed.ncbi.nlm.nih.gov/352355/352355 10.1016/0006-291x(78)91268-8

[16] Subedi KP, Choi D, Kim I, Min B, Park C. Hsp31 of Escherichia coli K-12 is glyoxalase III. Mol Microbiol [Internet]. 2011 Aug [cited 2022 Jan 14];81(4):926–36. Available from: https://pubmed.ncbi.nlm.nih.gov/21696459/ doi: 10.1111/j.1365-2958.2011.07736.x 21696459

[17] Engqvist M, Drincovich MF, Flügge UI, Maurino VG. Two D-2-hydroxy-acid dehydrogenases in Arabidopsis thaliana with catalytic capacities to participate in the last reactions of the methylglyoxal and beta-oxidation pathways. J Biol Chem [Internet]. 2009 Sep 11 [cited 2022 Jan 14];284(37):25026–37. Available from: https://pubmed.ncbi.nlm.nih.gov/19586914/ doi: 10.1074/jbc.M109.021253 19586914 PMC2757207

[18] Yadav SK, Singla-Pareek SL, Ray M, Reddy MK, Sopory SK. Methylglyoxal levels in plants under salinity stress are dependent on glyoxalase I and glutathione. Biochem Biophys Res Commun [Internet]. 2005 Nov 11 [cited 2022 Jan 14];337(1):61–7. Available from: https://pubmed.ncbi.nlm.nih.gov/16176800/ doi: 10.1016/j.bbrc.2005.08.263 16176800

[19] Singla-Pareek SL, Reddy MK, Sopory SK. Genetic engineering of the glyoxalase pathway in tobacco leads to enhanced salinity tolerance. Proc Natl Acad Sci U S A [Internet]. 2003 Dec 9 [cited 2022 Jan 14];100(25):14672–7. Available from: https://pubmed.ncbi.nlm.nih.gov/14638937/ doi: 10.1073/pnas.2034667100 14638937 PMC299757

[20] Ghosh A, Pareek A, Sopory SK, Singla-Pareek SL. A glutathione responsive rice glyoxalase II, OsGLYII-2, functions in salinity adaptation by maintaining better photosynthesis efficiency and anti-oxidant pool. Plant J [Internet]. 2014 Oct 1 [cited 2022 Jan 14];80(1):93–105. Available from: https://pubmed.ncbi.nlm.nih.gov/25039836/ doi: 10.1111/tpj.12621 25039836

[21] Tang F, Li R, Zhou Y, Wang S, Zhou Q, Ding Z, et al. Genome-Wide Identification of Cassava Glyoxalase I Genes and the Potential Function of MeGLYI-13 in Iron Toxicity Tolerance. Int J Mol Sci [Internet]. 2022 May 6 [cited 2022 Oct 4];23(9):5212. Available from: https://www.mdpi.com/1422-0067/23/9/5212/htm 35563603 10.3390/ijms23095212 PMC9104206

[22] Singla-Pareek SL, Yadav SK, Pareek A, Reddy MK, Sopory SK. Transgenic tobacco overexpressing glyoxalase pathway enzymes grow and set viable seeds in zinc-spiked soils. Plant Physiol [Internet]. 2006 [cited 2022 Jan 14];140(2):613–23. Available from: https://pubmed.ncbi.nlm.nih.gov/16384901/ doi: 10.1104/pp.105.073734 16384901 PMC1361328

[23] Ditt RF, Nester EW, Comai L. Plant gene expression response to Agrobacterium tumefaciens. Proc Natl Acad Sci U S A. 2001;98(19):10954–9. doi: 10.1073/pnas.191383498 11535836 PMC58580

[24] Zhou B, Peng K, Chu Z, Wang S, Zhang Q. The defense-responsive genes showing enhanced and repressed expression after pathogen infection in rice (Oryza sativa L.). Sci China Ser C, Life Sci [Internet]. 2002 [cited 2022 Jan 14];45(5):449–67. Available from: https://pubmed.ncbi.nlm.nih.gov/18759033/ doi: 10.1360/02yc9050 18759033

[25] FAOSTAT. Crops [Internet]. [cited 2022 Feb 23]. https://www.fao.org/faostat/en/#data/QCL

[26] Mustafiz A, Singh AK, Pareek A, Sopory SK, Singla-Pareek SL. Genome-wide analysis of rice and Arabidopsis identifies two glyoxalase genes that are highly expressed in abiotic stresses. Funct Integr Genomics. 2011;11(2):293–305. doi: 10.1007/s10142-010-0203-2 21213008

[27] Goodstein DM, Shu S, Howson R, Neupane R, Hayes RD, Fazo J, et al. Phytozome: A comparative platform for green plant genomics. Nucleic Acids Res. 2012;40(D1):1178–86. doi: 10.1093/nar/gkr944 22110026 PMC3245001

[28] Finn RD, Coggill P, Eberhardt RY, Eddy SR, Mistry J, Mitchell AL, et al. The Pfam protein families database: towards a more sustainable future. Nucleic Acids Res [Internet]. 2016 Jan 4 [cited 2021 Jul 24];44(D1):D279–85. Available from: https://academic.oup.com/nar/article/44/D1/D279/2503120 doi: 10.1093/nar/gkv1344 26673716 PMC4702930

[29] Blum M, Chang HY, Chuguransky S, Grego T, Kandasaamy S, Mitchell A, et al. The InterPro protein families and domains database: 20 years on. Nucleic Acids Res. 2021;49(D1):D344–54. doi: 10.1093/nar/gkaa977 33156333 PMC7778928

[30] Jones P, Binns D, Chang HY, Fraser M, Li W, McAnulla C, et al. InterProScan 5: Genome-scale protein function classification. Bioinformatics. 2014;30(9):1236–40. doi: 10.1093/bioinformatics/btu031 24451626 PMC3998142

[31] Letunic I, Bork P. 20 years of the SMART protein domain annotation resource. Nucleic Acids Res [Internet]. 2018 Jan 4 [cited 2022 Oct 4];46(D1):D493–6. Available from: https://academic.oup.com/nar/article/46/D1/D493/4429069 doi: 10.1093/nar/gkx922 29040681 PMC5753352

[32] Letunic I, Khedkar S, Bork P. SMART: recent updates, new developments and status in 2020. Nucleic Acids Res [Internet]. 2021 Jan 8 [cited 2022 Oct 4];49(D1):D458–60. Available from: https://academic.oup.com/nar/article/49/D1/D458/5940513 doi: 10.1093/nar/gkaa937 33104802 PMC7778883

[33] Gasteiger E, Hoogland C, Gattiker A, Duvaud S, Wilkins MR, Appel RD, et al. The Proteomics Protocols Handbook. Proteomics Protoc Handb. 2005;571–608.

[34] Chou K-C, Shen H-B. Cell-PLoc: a package of Web servers for predicting subcellular localization of proteins in various organisms. Nat Protoc [Internet]. 2008 Feb 17 [cited 2021 Jul 24];3(2):153–62. Available from: https://pubmed.ncbi.nlm.nih.gov/18274516/ doi: 10.1038/nprot.2007.494 18274516

[35] Suyama M, Torrents D, Bork P. PAL2NAL: Robust conversion of protein sequence alignments into the corresponding codon alignments. Nucleic Acids Res. 2006;34(WEB. SERV. ISS.):609–12. doi: 10.1093/nar/gkl315 16845082 PMC1538804

[36] Waterhouse AM, Procter JB, Martin DMA, Clamp M, Barton GJ. Jalview Version 2-A multiple sequence alignment editor and analysis workbench. Bioinformatics. 2009;25(9):1189–91. doi: 10.1093/bioinformatics/btp033 19151095 PMC2672624

[37] Kumar S, Stecher G, Li M, Knyaz C, Tamura K. MEGA X: Molecular Evolutionary Genetics Analysis across Computing Platforms. Battistuzzi FU, editor. Mol Biol Evol [Internet]. 2018 Jun 1;35(6):1547–9. Available from: https://academic.oup.com/mbe/article/35/6/1547/4990887 doi: 10.1093/molbev/msy096 29722887 PMC5967553

[38] Letunic I, Bork P. Interactive Tree Of Life (iTOL) v5: an online tool for phylogenetic tree display and annotation. Nucleic Acids Res [Internet]. 2021 Jul 2 [cited 2021 Jul 24];49(W1):W293–6. Available from: https://academic.oup.com/nar/article/49/W1/W293/6246398 doi: 10.1093/nar/gkab301 33885785 PMC8265157

[39] Hu B, Jin J, Guo A-Y, Zhang H, Luo J, Gao G. GSDS 2.0: an upgraded gene feature visualization server. Bioinformatics [Internet]. 2015 Apr 15 [cited 2021 Jul 24];31(8):1296–7. Available from: https://academic.oup.com/bioinformatics/article/31/8/1296/213025 doi: 10.1093/bioinformatics/btu817 25504850 PMC4393523

[40] Waterhouse A, Bertoni M, Bienert S, Studer G, Tauriello G, Gumienny R, et al. SWISS-MODEL: homology modelling of protein structures and complexes. Nucleic Acids Res [Internet]. 2018 Jul 2;46(W1):W296–303. Available from: https://academic.oup.com/nar/article/46/W1/W296/5000024 doi: 10.1093/nar/gky427 29788355 PMC6030848

[41] Meng EC, Goddard TD, Pettersen EF, Couch GS, Pearson ZJ, Morris JH, et al. UCSF ChimeraX: Tools for structure building and analysis. Protein Sci [Internet]. 2023 Nov 20;32(11). Available from: https://onlinelibrary.wiley.com/doi/10.1002/pro.4792 37774136 10.1002/pro.4792 PMC10588335

[42] Hruz T, Laule O, Szabo G, Wessendorp F, Bleuler S, Oertle L, et al. Genevestigator V3: A Reference Expression Database for the Meta-Analysis of Transcriptomes. Adv Bioinformatics. 2008 Jul 8;2008:1–5. doi: 10.1155/2008/420747 19956698 PMC2777001

[43] Howe EA, Sinha R, Schlauch D, Quackenbush J. RNA-Seq analysis in MeV. Bioinformatics [Internet]. 2011 Nov 15 [cited 2021 Jul 24];27(22):3209–10. Available from: https://academic.oup.com/bioinformatics/article/27/22/3209/194267 doi: 10.1093/bioinformatics/btr490 21976420 PMC3208390

[44] Saeed AI, Bhagabati NK, Braisted JC, Liang W, Sharov V, Howe EA, et al. [9] TM4 Microarray Software Suite. Methods Enzymol. 2006 Jan 1;411:134–93. 16939790 10.1016/S0076-6879(06)11009-5

[45] Lescot M. PlantCARE, a database of plant cis-acting regulatory elements and a portal to tools for in silico analysis of promoter sequences. Nucleic Acids Res [Internet]. 2002 Jan 1 [cited 2021 Aug 4];30(1):325–7. Available from: https://pubmed.ncbi.nlm.nih.gov/11752327/ doi: 10.1093/nar/30.1.325 11752327 PMC99092

[46] Bai S, Wang X, Guo M, Cheng G, Khan A, Yao W, et al. Selection and Evaluation of Reference Genes for Quantitative Real-Time PCR in Tomato (Solanum lycopersicum L.) Inoculated with Oidium neolycopersici. Agronomy [Internet]. 2022 Dec 14;12(12):3171. Available from: https://www.mdpi.com/2073-4395/12/12/3171

[47] Islam MS, Hasan MS, Hasan MN, Prodhan SH, Islam T, Ghosh A. Genome-wide identification, evolution, and transcript profiling of Aldehyde dehydrogenase superfamily in potato during development stages and stress conditions. Sci Rep [Internet]. 2021;11(1):1–17. Available from: doi: 10.1038/s41598-021-97691-9 34521910 PMC8440639

[48] Bradford MM. A rapid and sensitive method for the quantitation of microgram quantities of protein utilizing the principle of protein-dye binding. Anal Biochem. 1976 May 7;72(1–2):248–54. doi: 10.1006/abio.1976.9999 942051

[49] Bhowal B, Singla-Pareek SL, Sopory SK, Kaur C. From methylglyoxal to pyruvate: a genome-wide study for the identification of glyoxalases and D-lactate dehydrogenases in Sorghum bicolor. BMC Genomics [Internet]. 2020 Dec 10;21(1):145. Available from: https://bmcgenomics.biomedcentral.com/articles/10.1186/s12864-020-6547-7 32041545 10.1186/s12864-020-6547-7 PMC7011430

[50] Li WH, Gojobori T, Nei M. Pseudogenes as a paradigm of neutral evolution. Nat 1981 2925820 [Internet]. 1981 Jul 1 [cited 2022 Jan 17];292(5820):237–9. Available from: https://www.nature.com/articles/292237a0 doi: 10.1038/292237a0 7254315

[51] Kosakovsky Pond SL, Frost SDW. Not So Different After All: A Comparison of Methods for Detecting Amino Acid Sites Under Selection. Mol Biol Evol [Internet]. 2005 May 1 [cited 2022 Jan 17];22(5):1208–22. Available from: https://academic.oup.com/mbe/article/22/5/1208/1066893 doi: 10.1093/molbev/msi105 15703242

[52] Yang Z, Bielawski JR. Statistical methods for detecting molecular adaptation. Trends Ecol Evol [Internet]. 2000 [cited 2022 Jan 17];15(12):496. Available from: /pmc/articles/PMC7134603/ doi: 10.1016/s0169-5347(00)01994-7 11114436 PMC7134603

[53] Mustafiz A, Ghosh A, Tripathi AK, Kaur C, Ganguly AK, Bhavesh NS, et al. A unique Ni2+ -dependent and methylglyoxal-inducible rice glyoxalase i possesses a single active site and functions in abiotic stress response. Plant J. 2014;78(6):951–63. doi: 10.1111/tpj.12521 24661284

[54] Kaur C, Tripathi AK, Nutan KK, Sharma S, Ghosh A, Tripathi JK, et al. A nuclear-localized rice glyoxalase I enzyme, OsGLYI-8, functions in the detoxification of methylglyoxal in the nucleus. Plant J. 2017 Feb;89(3):565–76. doi: 10.1111/tpj.13407 27797431

[55] Ridderström M, Saccucci F, Hellman U, Bergman T, Principato G, Mannervik B. Molecular cloning, heterologous expression, and characterization of human glyoxalase II. J Biol Chem [Internet]. 1996 Jan 5 [cited 2022 Oct 25];271(1):319–23. Available from: http://www.jbc.org/article/S0021925818953421/fulltext doi: 10.1074/jbc.271.1.319 8550579

[56] Cameron AD, Ridderström M, Olin B, Mannervik B. Crystal structure of human glyoxalase II and its complex with a glutathione thiolester substrate analogue. Structure [Internet]. 1999 Sep 15 [cited 2022 Oct 2];7(9):1067–78. Available from: http://www.cell.com/article/S0969212699801749/fulltext 10508780 10.1016/s0969-2126(99)80174-9

[57] Kaur C, Vishnoi A, Ariyadasa TU, Bhattacharya A, Singla-Pareek SL, Sopory SK. Episodes of horizontal gene-transfer and gene-fusion led to co-existence of different metal-ion specific glyoxalase i. Sci Rep. 2013;3(Ii):1–10. doi: 10.1038/srep03076 24220130 PMC3826101

[58] Ridderström M, Cameron AD, Jones TA, Mannervik B. Involvement of an active-site Zn2+ ligand in the catalytic mechanism of human glyoxalase I. J Biol Chem [Internet]. 1998 Aug 21 [cited 2022 Oct 25];273(34):21623–8. Available from: http://www.jbc.org/article/S0021925818488291/fulltext doi: 10.1074/jbc.273.34.21623 9705294

[59] Kaur C, Mustafiz A, Sarkar AK, Ariyadasa TU, Singla-Pareek SL, Sopory SK. Expression of abiotic stress inducible ETHE1-like protein from rice is higher in roots and is regulated by calcium. Physiol Plant [Internet]. 2014 Sep 1 [cited 2022 Jan 18];152(1):1–16. Available from: https://onlinelibrary.wiley.com/doi/full/10.1111/ppl.12147 24410953 10.1111/ppl.12147

[60] Ghosh A. Genome-wide identification of glyoxalase genes in medicago truncatula and their expression profiling in response to various developmental and environmental stimuli. Front Plant Sci. 2017;8(June). doi: 10.3389/fpls.2017.00836 28620395 PMC5452422

[61] Lin J, Prahlad J, Wilson MA. Conservation of oxidative protein stabilization in an insect homologue of the parkinsonism-associated protein DJ-1. Biochemistry [Internet]. 2012 May 8 [cited 2022 Jan 21];51(18):3799. Available from: /pmc/articles/PMC3348460/22515803 10.1021/bi3003296 PMC3348460

[62] Jin S, Chen X, Yang J, Ding J. Lactate dehydrogenase D is a general dehydrogenase for D-2-hydroxyacids and is associated with D-lactic acidosis. Nat Commun. 2023;14(1):1–13. 37863926 10.1038/s41467-023-42456-3 PMC10589216

[63] Du L, Ma Z, Mao H. Duplicate Genes Contribute to Variability in Abiotic Stress Resistance in Allopolyploid Wheat. Plants [Internet]. 2023 Jun 28;12(13):2465. Available from: https://www.mdpi.com/2223-7747/12/13/2465 doi: 10.3390/plants12132465 37447026 PMC10346836

[64] Kumar B, Kaur C, Pareek A, Sopory SK, Singla-Pareek SL. Tracing the evolution of plant glyoxalase iii enzymes for structural and functional divergence. Antioxidants. 2021 May 1;10(5). doi: 10.3390/antiox10050648 33922426 PMC8170915

[65] Jana GA, Krishnamurthy P, Kumar PP, Yaish MW. Functional characterization and expression profiling of glyoxalase III genes in date palm grown under abiotic stresses. Physiol Plant [Internet]. 2021 Jun 1 [cited 2022 Oct 10];172(2):780–94. Available from: https://squ.pure.elsevier.com/en/publications/functional-characterization-and-expression-profiling-of-glyoxalas doi: 10.1111/ppl.13239 33034392

[66] Liu S, Liu W, Lai J, Liu Q, Zhang W, Chen Z, et al. OsGLYI3, a glyoxalase gene expressed in rice seed, contributes to seed longevity and salt stress tolerance. Plant Physiol Biochem. 2022 Jul 15;183:85–95. doi: 10.1016/j.plaphy.2022.04.028 35569169

[67] Ghosh A, Mustafiz A, Pareek A, Sopory SK, Singla-Pareek SL. Glyoxalase III enhances salinity tolerance through reactive oxygen species scavenging and reduced glycation. Physiol Plant. 2022 May 1;174(3). 35483971 10.1111/ppl.13693

[68] Gambhir P, Singh V, Raghuvanshi U, Parida AP, Pareek A, Roychowdhury A, et al. A glutathione-independent DJ-1/PfpI domain-containing tomato glyoxalaseIII2, SlGLYIII2, confers enhanced tolerance under salt and osmotic stresses. Plant Cell Environ [Internet]. 2023 Feb 26;46(2):518–48. Available from: https://onlinelibrary.wiley.com/doi/10.1111/pce.14493 36377315 10.1111/pce.14493

[69] Mohanan MV, Thelakat Sasikumar SP, Jayanarayanan AN, Selvarajan D, Ramanathan V, Shivalingamurthy SG, et al. Transgenic sugarcane overexpressing Glyoxalase III improved germination and biomass production at formative stage under salinity and water-deficit stress conditions. 3 Biotech [Internet]. 2024 Feb 23;14(2):52. Available from: https://link.springer.com/10.1007/s13205-023-03856-w 38274846 10.1007/s13205-023-03856-w PMC10805895

