# Supplementary material for: Methylglyoxal detoxifying gene families in tomato: Genome-wide identification, evolution, functional prediction, and transcript profiling
Source: PLoS One. 2024 Jun 12;19(6):e0304039. doi: 10.1371/journal.pone.0304039 (PMC11168688; doi:10.1371/journal.pone.0304039)
Supplement: S1 Table — (DOCX) [file pone.0304039.s001.docx]

**S1 Table.** Primer sequences of selected *SlGLY* and *SlDLDH* genes were used in the study.

| Sl No | Primer name | Primer Sequence (5’-3’) | Product (bp) |
| --- | --- | --- | --- |
| 1 | SlGLYI-2FOR | CCGTTGAACGTACTGCTTGG | 122 |
| 2 | SlGLYI-2REV | GCCACGTGGTTCTGAATTTCC |  |
| 3 | SlGLYI-3FOR | TGGAACTGTCACAAGGGAGC | 142 |
| 4 | SlGLYI-3REV | CACACGAAGCATCACTTGGC |  |
| 5 | SlGLYI-6A FOR | ACCCTCTCTTTTCACTTTCCG | 95 |
| 6 | SlGLYI-6A REV | TCTTTCCTCGGGGATGTCAC |  |
| 7 | SlGLYI-6B FOR | GGATGCGGTATCATTGTGCC | 80 |
| 8 | SlGLYI-6B REV | TGTGACTGGGTGATGACGAG |  |
| 9 | SlGLYII-1A FOR | TGTGTATGGCCTGGCATGAG | 93 |
| 10 | SlGLYII-1A REV | ACGTACAGTCTTGAACGGCA |  |
| 11 | SlGLYII-1B FOR | CGATGTTGACCGAAGCCTCT | 142 |
| 12 | SlGLYII-1B REV | CACCATGCCAGCAAACTCAG |  |
| 13 | SlGLYII-3A FOR | AAAGGGTTCACCGTCAGCTC | 134 |
| 14 | SlGLYII-3A REV | GGAACTGCGACGTCCATCAT |  |
| 15 | SlGLYII-3B FOR | GCAACTCCCGGTCATACGTT | 136 |
| 16 | SlGLYII-3B REV | TTCCACCCTGAAAGTCCGTC |  |
| 17 | SlDJ-1A FOR | CGAGTATGCTGTTGCGTTGG | 105 |
| 18 | SlDJ-1A REV | ACATACGCAGTTCCTCCAGC |  |
| 19 | SlDJ-1C FOR | AACTACTACTGCCGCTTCCG | 123 |
| 20 | SlDJ-1C REV | ACTGTTACCTCTGCACCAGC |  |
| 21 | SlDJ-1D FOR | AATCTGCCATGGGCAACAGA | 145 |
| 22 | SlDJ-1D REV | GTCCGTGAAGCAACGGTCTA |  |
| 23 | SlDLDH-1 FOR | AGGTCTGAGGAGGAGGTGTC | 123 |
| 24 | SlDLDH-1 REV | GCAAACACCACCATAGGGAGA |  |
| 25 | SlDLDH-2 FOR | ATTAGTGCCGAACATGGCCT | 158 |
| 26 | SlDLDH-2 REV | TCTGTAAAAAGAGAAGGCGGG |  |
| 27 | SlDLDH-3 FOR | AGCACGTGGAGATGGGAATC | 142 |
| 28 | SlDLDH-3 REV | ACAAGAGAGGCCAAACAGAAC |  |
| 29 | SlDLDH-4 FOR | ACACAAAAGGCAGGGTGACA | 130 |
| 30 | SlDLDH-4 REV | CCAAAGCTCAAGTTGTCGGC |  |
| 31 | SlEF1α_FOR | ACAGGCGTTCAGGTAAGGAA | 120 |
| 32 | SlEF1α_REV | GAGGGTATTCAGCAAAGGTCTC |  |
