# Supplementary material for: Methylglyoxal detoxifying gene families in tomato: Genome-wide identification, evolution, functional prediction, and transcript profiling
Source: PLoS One. 2024 Jun 12;19(6):e0304039. doi: 10.1371/journal.pone.0304039 (PMC11168688; doi:10.1371/journal.pone.0304039)
Supplement: S2 Table — (DOCX) [file pone.0304039.s002.docx]

**S2 Table.** Gene duplication event between tomato glyoxalases and D-lactate dehydrogenase genes

| Locus 1 | Locus 2 | d_s_ | d_n_ | d_n_/ d_s_ | Duplication time (Mya)  T= d_s_/2* λ | Duplication type |
| --- | --- | --- | --- | --- | --- | --- |
| SlGLYI-4A | SlGLYI-7A | 1.137 | 0.163 | 0.1186 | 37.9 | Segmental |
| SlGLYII-3A | SlGLYII-3B | 0.708 | 0.131 | 0.1863 | 23.6 | Segmental |
| SlDLDH-3 | SlDLDH-4 | 0.885 | 0.125 | 0.1412 | 29.5 | Segmental |
