# Supplementary material for: Methylglyoxal detoxifying gene families in tomato: Genome-wide identification, evolution, functional prediction, and transcript profiling
Source: PLoS One. 2024 Jun 12;19(6):e0304039. doi: 10.1371/journal.pone.0304039 (PMC11168688; doi:10.1371/journal.pone.0304039)
Supplement: S3 Table — (DOCX) [file pone.0304039.s003.docx]

**S3 Table.** Information on domain organisation of SlDJ-1 proteins for the prediction of enzymatic activity

| Protein | Protein domain | | | | | | Conserved catalytic site | | | Predicted enzyme activity |
| --- | --- | --- | --- | --- | --- | --- | --- | --- | --- | --- |
|  | N- Terminal DJ-1 | | | C– terminal DJ-1 | | | Asp/Glu | Cys | Tyr/His |  |
|  | Start | End | Length | Start | End | Length |  |  |  |  |
| SlDJ-1A | 56 | 249 | 194 | 304 | 465 | 162 | + | + | + | Active |
| SlDJ-1C | 17 | 181 | 165 | 221 | 381 | 161 | + | + | + | Active |
| SlDJ-1D | 5 | 187 | 183 | 198 | 380 | 183 | + | + | + | Active |
