# Supplementary material for: Methylglyoxal detoxifying gene families in tomato: Genome-wide identification, evolution, functional prediction, and transcript profiling
Source: PLoS One. 2024 Jun 12;19(6):e0304039. doi: 10.1371/journal.pone.0304039 (PMC11168688; doi:10.1371/journal.pone.0304039)
Supplement: S4 Table — (DOCX) [file pone.0304039.s004.docx]

**S4 Table.** Information on domain organisation of SlDLDH proteins for the prediction of enzymatic activity

| Protein | Protein Domains | | | | | | Predicted enzyme activity |
| --- | --- | --- | --- | --- | --- | --- | --- |
|  | FAD_binding_4 | | | FAD_oxidase_C | | |  |
|  | Start | End | Length | Start | End | Length |  |
| SlDLDH-1 | 150 | 287 | 138 | 323 | 564 | 242 | + |
| SlDLDH-2 | 139 | 277 | 139 | 314 | 557 | 244 | + |
| SlDLDH-3 | 122 | 257 | 136 | - | - | - | + |
| SlDLDH-4 | 86 | 200 | 115 | - | - | - | + |
