# Supplementary material for: Methylglyoxal detoxifying gene families in tomato: Genome-wide identification, evolution, functional prediction, and transcript profiling
Source: PLoS One. 2024 Jun 12;19(6):e0304039. doi: 10.1371/journal.pone.0304039 (PMC11168688; doi:10.1371/journal.pone.0304039)
Supplement: S6 Table — (DOCX) [file pone.0304039.s006.docx]

**S5 Table.** The presence of cis-regulatory elements in the promoter region in *SlGLY* and *SlDLDH* genes

|  | ABRE | LTR | TGACG-motif | MBS | WUN-motif | ERE | AAGAA-motif | GARE motif | AuxRR-core | TC-rich repeats | TCA element | box w | Total |
| --- | --- | --- | --- | --- | --- | --- | --- | --- | --- | --- | --- | --- | --- |
| SlGLYI-1 | 2 | 1 | 1 |  |  |  |  | 1 |  |  |  |  | 5 |
| SlGLYI-2 | 18 | 4 | 6 | 3 |  | 19 |  |  | 1 | 5 | 1 | 7 | 64 |
| SlGLYI-3 | 5 | 3 | 1 |  | 3 | 3 | 8 |  |  | 2 | 2 |  | 27 |
| SlGLYI-4A | 5 |  |  | 1 | 2 | 2 |  |  |  | 1 |  |  | 11 |
| SlGLYI-4B | 5 |  | 4 | 1 | 2 | 2 | 1 |  |  |  |  |  | 15 |
| SlGLYI-6A | 7 | 1 | 4 | 3 | 2 | 1 |  | 1 | 1 | 2 | 1 | 1 | 24 |
| SlGLYI-6B | 7 | 1 |  | 9 | 3 | 1 |  | 3 | 2 | 2 | 1 | 3 | 32 |
| SlGLYI-7A | 1 |  |  | 1 |  | 1 |  |  |  |  | 2 | 1 | 6 |
| SlGLYI-7B | 5 |  |  |  | 1 | 8 |  |  |  |  | 4 | 3 | 21 |
| SlGLYI-7C | 1 |  | 0 | 0 |  |  |  |  |  |  |  |  | 1 |
| SlGLYI-8 | 8 |  |  |  | 2 |  |  |  |  |  |  |  | 10 |
| SlGLYI-9 | 3 | 1 | 3 |  |  | 2 |  |  | 1 |  | 1 | 1 | 12 |
| SlGLYI-11 | 4 | 1 | 6 | 2 | 1 | 6 |  |  |  |  |  | 1 | 21 |
| SlGLYII-1A | 4 | 1 | 7 | 12 | 2 | 4 |  | 3 | 1 | 1 | 6 | 4 | 45 |
| SlGLYII-1B | 7 | 2 |  | 7 | 5 | 3 |  |  | 1 | 2 | 5 | 3 | 35 |
| SlGLYII-3A | 2 | 1 | 4 | 7 | 2 | 1 |  |  | 1 | 2 | 3 | 2 | 25 |
| SlGLYII-3B | 5 | 2 | 1 | 4 |  | 1 |  |  |  | 2 | 2 | 1 | 18 |
| SlDJ-1A | 6 | 3 | 2 | 2 |  | 3 |  |  |  |  | 2 | 3 | 21 |
| SlDJ-1C | 3 | 2 | 3 | 4 | 2 | 1 |  | 2 |  |  | 1 |  | 18 |
| SlDJ-1D | 5 |  | 1 | 2 | 2 |  |  |  |  | 4 | 2 | 3 | 19 |
| SlDLDH-1 | 29 | 8 | 30 | 27 | 14 | 13 |  | 9 | 6 | 7 | 8 | 24 | 175 |
| SlDLDH-2 | 4 | 1 | 10 | 5 | 1 | 6 |  |  | 1 | 5 | 3 | 1 | 37 |
| SlDLDH-3 | 2 |  | 2 | 1 |  |  |  |  | 1 |  | 2 |  | 8 |
| SlDLDH-4 | 2 |  |  | 5 | 2 |  |  |  |  |  | 3 |  | 12 |
