# Supplementary figures and images for: Methylglyoxal detoxifying gene families in tomato: Genome-wide identification, evolution, functional prediction, and transcript profiling
Source: PLoS One. 2024 Jun 12;19(6):e0304039. doi: 10.1371/journal.pone.0304039 (PMC11168688; doi:10.1371/journal.pone.0304039)

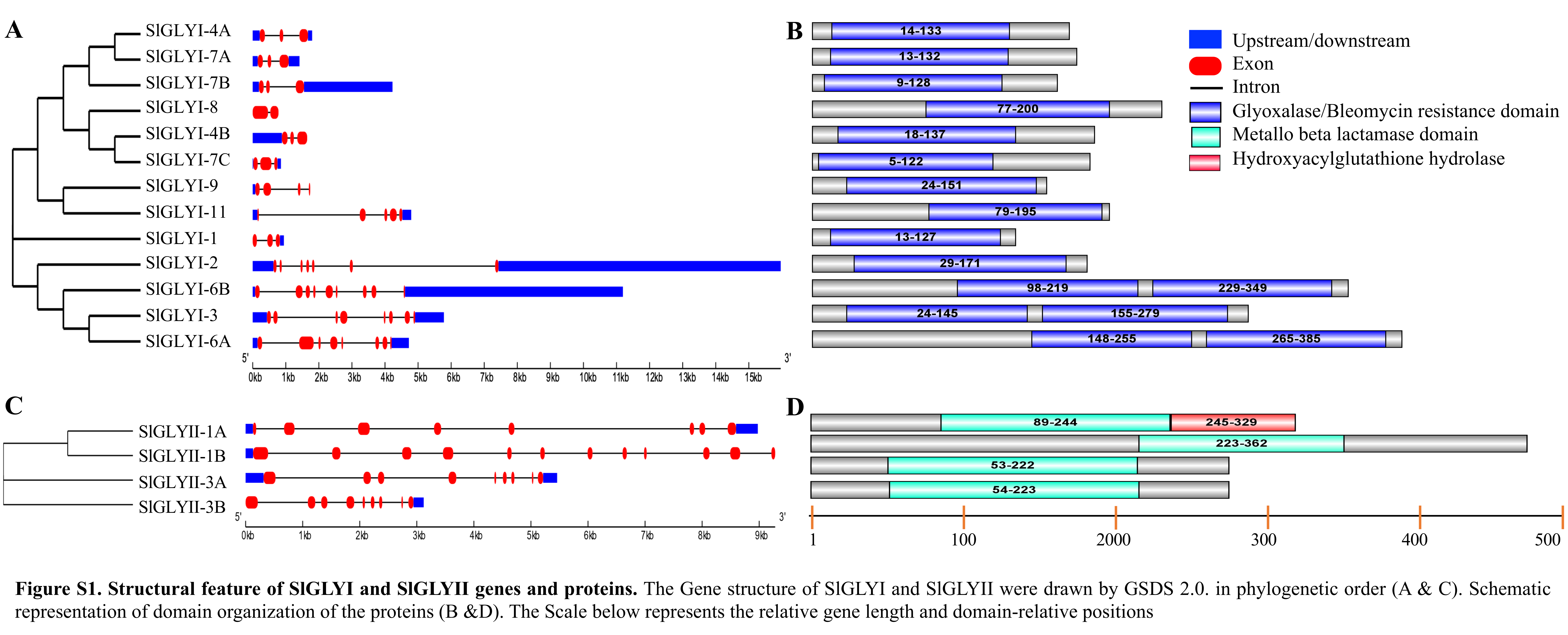

Supplement: S1 Fig — (TIF) [file pone.0304039.s008.tif]

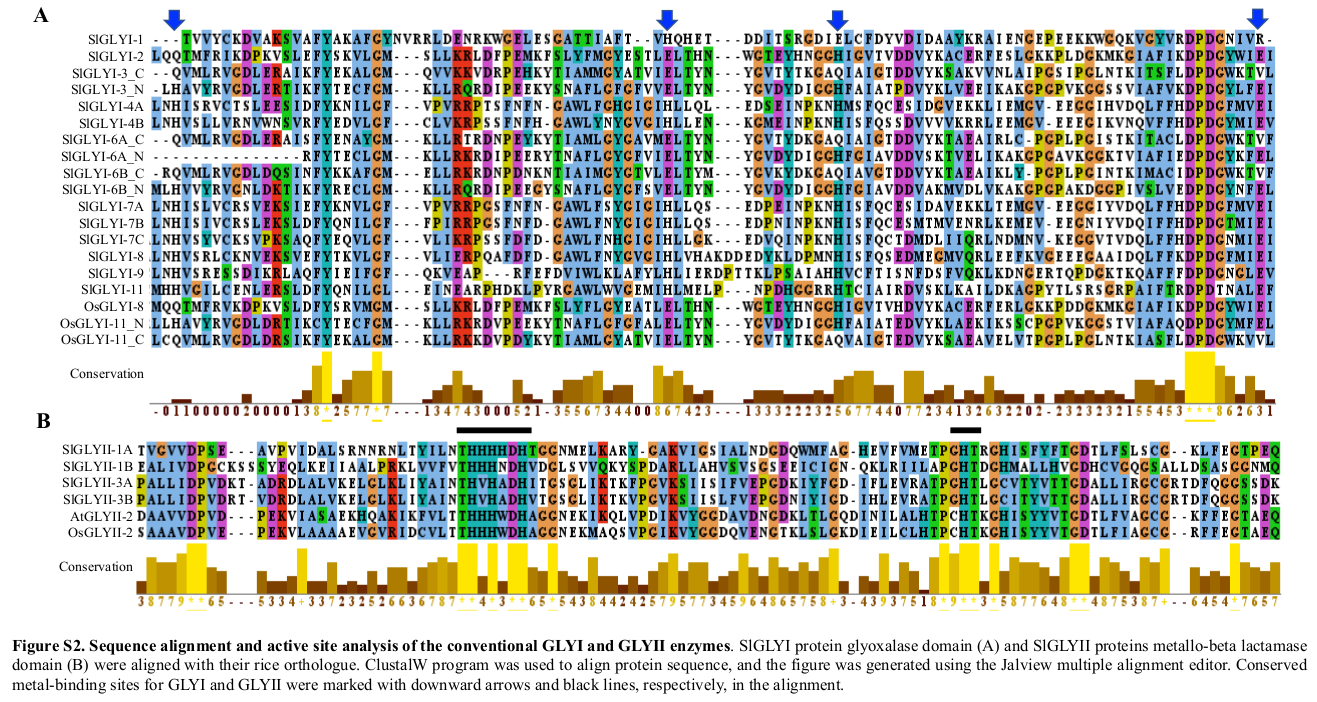

Supplement: S2 Fig — (TIF) [file pone.0304039.s009.tif]

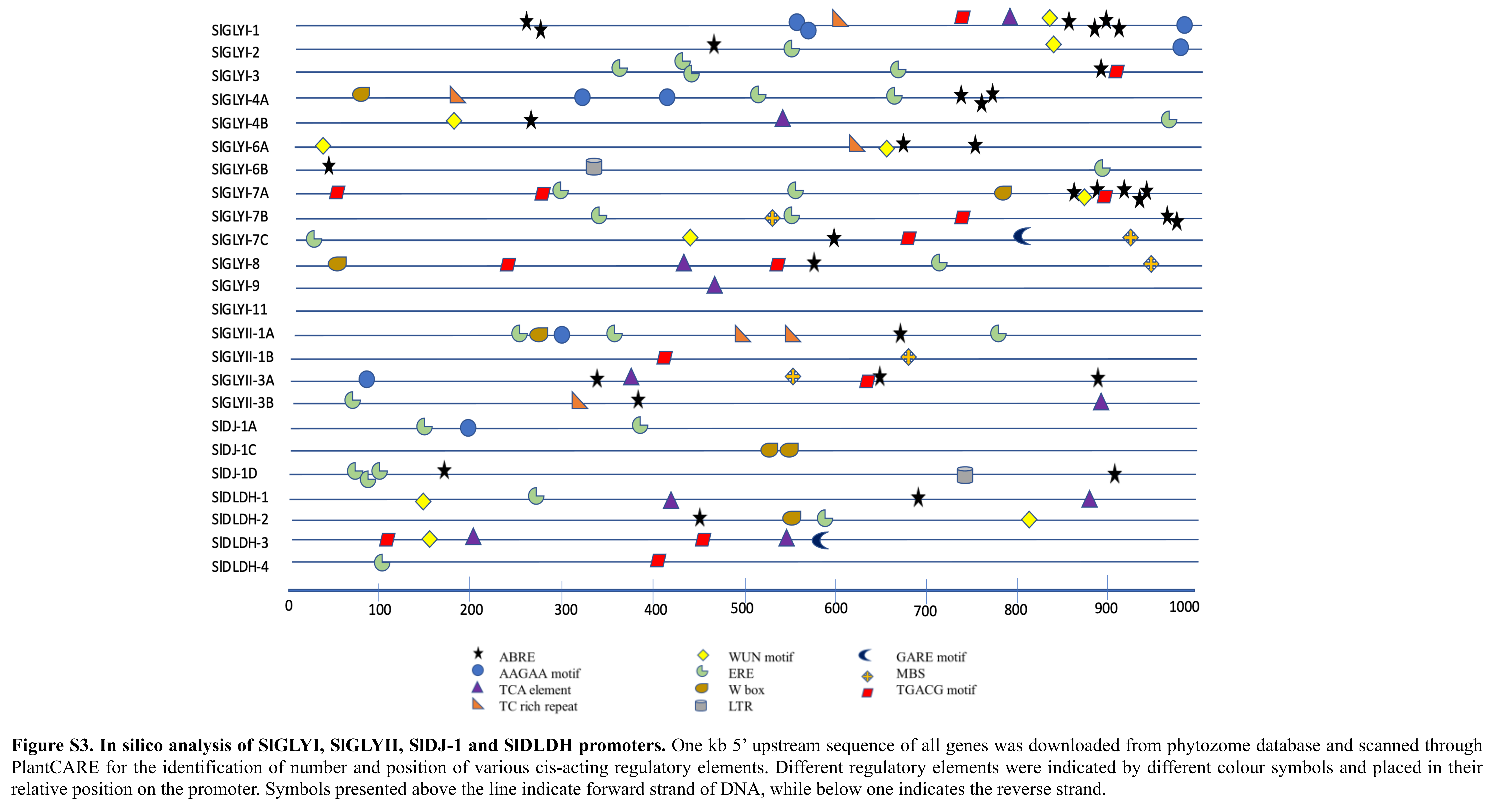

Supplement: S3 Fig — (TIF) [file pone.0304039.s010.tif]
